# Genomic meta-analysis of the interplay between 3D chromatin organization and gene expression programs under basal and stress conditions

**DOI:** 10.1186/s13072-018-0220-2

**Published:** 2018-08-29

**Authors:** Idan Nurick, Ron Shamir, Ran Elkon

**Affiliations:** 1 The Blavatnik School of Computer Science, Tel Aviv University, 69978 Tel Aviv, Israel; 2 Department of Human Molecular Genetics and Biochemistry, Sackler School of Medicine, Tel Aviv University, 69978 Tel Aviv, Israel

**Keywords:** 3D genome organization, Gene regulation, Meta-analysis, A/B compartments, Enhancer–promoter interactions

## Abstract

**Background:**

Our appreciation of the critical role of the genome’s 3D organization in gene regulation is steadily increasing. Recent 3C-based deep sequencing techniques elucidated a hierarchy of structures that underlie the spatial organization of the genome in the nucleus. At the top of this hierarchical organization are chromosomal territories and the megabase-scale A/B compartments that correlate with transcriptional activity within cells. Below them are the relatively cell-type-invariant topologically associated domains (TADs), characterized by high frequency of physical contacts between loci within the same TAD, and are assumed to function as regulatory units. Within TADs, chromatin loops bring enhancers and target promoters to close spatial proximity. Yet, we still have only rudimentary understanding how differences in chromatin organization between different cell types affect cell-type-specific gene expression programs that are executed under basal and challenged conditions.

**Results:**

Here, we carried out a large-scale meta-analysis that integrated Hi–C data from thirteen different cell lines and dozens of ChIP-seq and RNA-seq datasets measured on these cells, either under basal conditions or after treatment. Pairwise comparisons between cell lines demonstrate a strong association between modulation of A/B compartmentalization, differential gene expression and transcription factor (TF) binding events. Furthermore, integrating the analysis of transcriptomes of different cell lines in response to various challenges, we show that A/B compartmentalization of cells under basal conditions significantly correlates not only with gene expression programs and TF binding profiles that are active under the basal condition but also with those induced in response to treatment. Yet, in pairwise comparisons between different cell lines, we find that a large portion of differential TF binding and gene induction events occur in genomic loci assigned to A compartment in both cell types, underscoring the role of additional critical factors in determining cell-type-specific transcriptional programs.

**Conclusions:**

Our results further indicate the role of dynamic genome organization in regulation of differential gene expression between different cell types and the impact of intra-TAD enhancer–promoter interactions that are established under basal conditions on both the basal and treatment-induced gene expression programs.

**Electronic supplementary material:**

The online version of this article (10.1186/s13072-018-0220-2) contains supplementary material, which is available to authorized users.

## Background

3C-based methods measure the frequency of physical interactions between any pair of genomic loci as a proxy for their spatial proximity. These novel technologies are shedding light on the principles of 3D organization of the genome in the nucleus and its relation to gene regulation [1–3]. A four-layer hierarchy of structures is emerging from these studies [4, 5]. At the top of this hierarchy are the chromosomes which are generally organized in a way that gene-dense chromosomes tend to be at the nuclear interior, whereas the more gene-poor chromosomes are found near the nuclear periphery [6]. In the next layer are megabase-scale genomic compartments that are either euchromatic, gene-rich, and transcriptionally active (called A compartments) or heterochromatic, gene-poor, and transcriptionally silent (called B compartments) [5, 7]. Spatially, the open (A-type) compartments cluster together in the nuclear interior, whereas the closed (B-type) compartments tend to cluster near the nuclear periphery [4]. These chromosomal compartments contain ~ 100 kb–1 Mb scale subunits called topologically associating domains (TADs). These are characterized by high frequency of interactions between loci located in the same domain, and much lower interaction rate between loci located in adjacent TADs [8, 9]. Unlike the A/B compartments, which associate with gene expression and therefore markedly vary between different cell types, TADs are largely invariant across different cell types and physiological conditions [7, 10]. At the bottom of the hierarchy are ~ 10 Kb–1 Mb chromatin-looping interactions, bringing enhancers (*E*) and promoters (*P*) that are located at high distance along the linear DNA sequence to close spatial proximity. Such E–P loops, a portion of which is cell type specific, mostly occur within TADs and unfrequently cross over TAD boundaries [4, 10]. The 3D organization of the genome has a pronounced cell-to-cell stochastic variability, and the snapshots obtained by 3C-based analyses are typically the result of averaging over a large ensemble of cells.

Our understanding of the roles that the 3D organization of the genome plays in gene regulation has markedly increased in recent years. It emerges that TADs serve as fundamental structural and regulatory building blocks of chromosomes that constrain and largely exclude physical interactions between genes and regulatory elements located in different TADs, while providing sufficiently dynamic local environment that is required for the establishment of intra-TAD E–P links [4, 8]. In line with the view of TADs as structural regulatory units, examination of the dynamic changes in genome 3D organization during differentiation of stem cells into six different linages showed that the regions that changed their A/B compartment mostly corresponded to a single or a series of adjacent TADs [11]. In addition, no significant changes in TAD boundaries were detected in a breast cancer cell line upon treatment with hormone, suggesting that TADs are also invariant under transient cell challenges [12]. Furthermore, this study found a statistically significant, though limited, number of TADs that behaved as discrete regulatory units where the majority of the genes inside them were either coordinately induced or repressed.

Intra-TAD E–P links are required for the implementation of transcriptional programs that establish and maintain cell identity and responses to environmental cues. How these regulatory interactions are modulated in response to transient perturbation is still not well understood. While some studies have shown that gene induction is accompanied by alterations of chromatin interactions and internal restructuring of TADs [12–14], unexpectedly, it was recently observed that the majority of TNF-α-responsive enhancers were already in contact with their target promoters before treatment [15]. Given that the transcriptional responses to various stresses show high level of cell-type specificity, these results suggest that intra-TAD interactions that are already in place in each cell type under basal conditions affect the spectrum of genes that are induced upon triggers in each cell type.

Here, we carried out a large-scale meta-analysis, integrating Hi–C data from 13 different cell lines and dozens of ChIP-seq and RNA-seq datasets recorded in the same cellular systems at basal conditions and in response to various treatments, to further elucidate the intricate interplay between the hierarchical 3D organization of the genome and gene regulation.

## Results

### Differences in gene expression between cell lines correlate with A/B compartmentalization

**Fig. 1:**
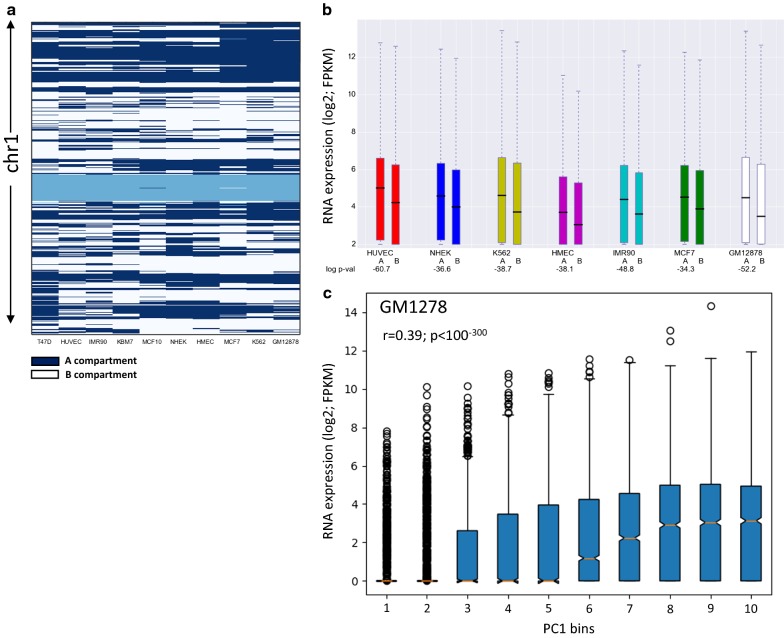
Chromosomal compartments and gene expression. **a** A/B partition of chromosome 1 for different cell lines based on Hi–C data in 100-kb resolution. Dark-blue and white indicate A and B compartments, respectively. Light blue indicates areas which Hi–C could not measure interactions for, e.g., centromeres. **b** Comparison of gene expression levels in A and B compartments for each cell line. *p* values (in log10) for the significance of the difference are indicated below each comparison (Wilcoxon’s test). For all cell lines, genes in A compartment are significantly more highly expressed than genes in B compartment. C. Correlation between the magnitude of the first principal component (PC1) and gene expression level in GM12878 cell line. Genes were divided into ten bins according to the magnitude of PC1, and distribution of expression levels was calculated for each bin

We first defined the higher-order organization of the genome into A/B compartments for 13 human cell lines for which Hi–C data are available (Additional file 1: Table S1). We normalized each Hi–C matrix and performed principal component analysis (PCA) for each intra-chromosomal matrix separately (“Methods” section). By definition, the A compartment is gene rich and is broadly associated with active transcription and epigenomic marks of open chromatin, while the B compartment is gene poor and associates with low transcriptional activity and condensed chromatin. Thus, for each chromosome separately, we used gene density to determine whether positive or negative values of the PC that represents the A/B compartmentalization (typically the first principal component, denoted PC1) corresponds to A compartment. (Centromeric regions were not included in the A/B partitions since no chromatin interactions are identified by Hi–C in these regions.) Table 1 summarizes the total genomic size and number of genes assigned to the A and B compartments in each cell line. As an example, Fig. 1a shows the partition into A/B compartments we obtained for chromosome 1 in the 13 cell lines. On average, 25% of the genome showed assignment to different compartments in pairwise comparisons between cell lines.

**Table 1:** Genomic size and number of genes assigned to A/B compartments

Cell line	A total size (Mbp)	# Genes in A	B total size (Mbp)	# Genes in B
GM12878	1322	15,184	1410	3958
K562	1376	15,401	1356	3741
HUVEC	1382	15,116	1350	4022
HMEC	1317	14,593	1415	4543
NHEK	1433	14,864	1300	4276
IMR90	1310	13,569	1423	5577
T47D	1372	14,114	1350	4979
MCF7	1384	15,056	1450	4758
MCF10	1386	15,090	1451	4772
LNCAP	1433	14,112	1299	5021
PC3	1395	13,341	1313	5692
KBM7	1301	14,506	1431	4631
PrEC	1327	13,994	1387	5041

**Fig. 2:**
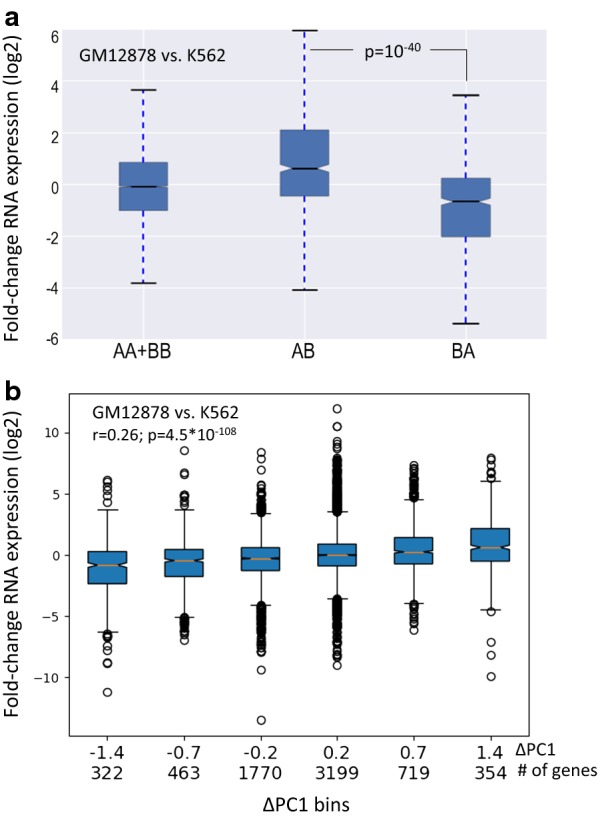
**a** Association between dynamic A/B compartmentalization and differential gene expression in the comparison between the GM12878 and K562 cell lines (AB: the set of genes that are located in compartment A in GM12878 and B in K562; BA: genes located in compartment B in GM12878 and A in K562). Genes in AB have significantly higher expression in GM12878, while genes in BA have higher expression in K562 (*p* value calculated using Wilcoxon’s test). **b** Correlation between the change in the magnitude of PC1 and change in expression level in the comparison between GM12878 and K562 cell lines. The range of ΔPC1 was divided into six bins, and the distribution of fold change in gene expression was calculated for each bin. Below each boxplot, the mean value of ΔPC1 in that bin and the number of genes assigned to it are indicated

As a first examination, per cell line, we confirmed that genes assigned to the A compartment are significantly more highly expressed than genes assigned to the B compartment (Fig. 1b). Furthermore, in a finer analysis, for all cell lines we observed a remarkable correlation between the magnitude of the PC that represents the A/B compartmentalization and gene expression level (Fig. 1c; Additional file 2: Fig. S1). Next, we tested for association between differences in A/B compartmentalization and gene expression across different cell lines. Specifically, for each pair of cell lines, we examined whether genes located in A compartment in one cell line and in B compartment in the other show higher expression in the former. Thus, for each pair of cell lines, we divided the genes into four sets—A in both cell lines (AA), B in both cell lines (BB), A in cell line 1 and B in cell line 2 (AB) and B in cell line 1 and A in cell line 2 (BA). We calculated gene expression ratios between cell lines 1 and 2 and compared the distribution of these ratios between the four gene sets. This analysis confirmed that genes in the AB set are significantly more highly expressed in cell line 1, while genes in the BA set show significantly higher expression in cell line 2 (Fig. 2a; Additional file 2: Fig. S2A). Here too, a finer quantitative analysis showed a highly significant correlation between the change in the magnitude of PC1 and the change in expression level (Fig. 2b; Additional file 2: Fig. S2B).

### Epigenetic differences between cell lines correlate with differences in A/B compartmentalization

As the A compartment is associated with open state of the chromatin we next systematically examined the association between A/B compartmentalization and TF binding. We analyzed 122 TF ChIP-seq datasets recorded by ENCODE for cell lines with Hi–C data (Additional file 1: Table S2). First, we measured TF binding site (TFBS) enrichment for the A compartment, for each cell line separately, by defining the A–B *density factor*, *D* (*D* > 1 (positive in log scale) implies that binding sites are enriched for the A compartment and *D* < 1 (negative in log scale) implies that binding sites are enriched for B compartment; “Methods” section). As expected, the chromatin-binding profile of all TFs in all examined cell lines showed a remarkable enrichment for the A compartment (Additional file 2: Fig. S3; see Additional file 1: Table S3 for one detailed example: CTCF).

**Fig. 3:**
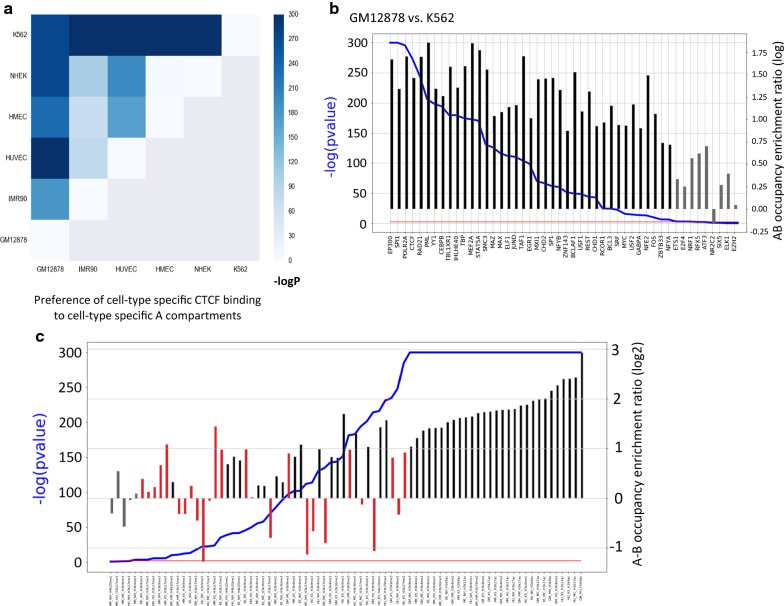
Cell-type-specific TF binding and histone modification events versus cell-type-specific compartments. **a** Relation between cell-type-specific A/B partition and CTCF binding sites for six cell lines. For all pair-wise comparisons, CTCF BSs specific to cell 1 (cell 2) showed a significant preference to AB (BA) genomic regions over BA (AB) regions. All *p* values are highly significant (–log10, Chi-square test). **b** Relation between cell-type-specific TFBSs and A/B compartments in the comparison between GM12878 and K562. For each of the 49 TFs we calculated the *AB occupancy enrichment ratio* as a measure for the preference of its cell-type-specific binding events to AB genomic regions over BA regions. Experiments are sorted by *p* value, and enrichment ratios are represented by bars. Red line: *p* value = 0.01 (statistically significant and nonsignificant results are presented by black and gray bars, respectively). **c** The same as B, but for histone modifications. The compared cell lines (using the first two letters of their names; e.g., GM = GM12878; K5 = K562) and the examined histone modification are indicated below each bar. The results for the repressive histone marks H3K27me3 and H3K9me3 are colored in red

Next, we examined whether A–B transitions between cell lines are reflected by TF binding profiles. For each pair of cell lines, numbered 1 and 2, we segmented the genome into four regions according to A/B assignment in the two cell lines as described above. For a given TF, we divided the TF binding sites into three groups: sites common to cell lines 1 and 2, sites detected only in cell line 1, and sites detected only in cell line 2. We then tested for a relationship between these two divisions. Specifically, we defined the A–B *occupancy enrichment ratio R* (see “Methods” section) to test whether cell-type-specific TFBSs occur more often in regions assigned as A compartment in the cell line where the binding occurs and as B in the other cell line than the opposite regions (that is, regions assigned as B-type in the cell line where the binding occurs and as A in the other one). Table 2a shows, as an example, the results obtained for CTCF binding sites in the comparison between the HMEC and HUVEC cell lines. As expected, we observed that CTCF BSs specific to HMEC (HUVEC) showed a significant preference to AB (BA) genomic regions over BA (AB) regions. As CTCF ChIP-seq data were available for six cell lines with Hi–C data, we could systematically carry out this comparison for this factor. In all pairwise tests, we observed a highly significant preference of CTCF cell-type-specific binding to cell-type-specific A over B regions (Fig. 3a). Yet, a large portion of cell-type-specific TFBSs were located in genomic regions that are assigned to A compartment in both cell lines (AA regions) (Table 2a), indicating that other factors in addition to A/B compartmentalization determine the TF-chromatin interaction profile in each cell type.

**Table 2:** Comparison of binding site and epigenetic mark occupancy in A/B compartments between two cell types

(A) CTCF; HMEC–HUVEC	AA	AB	BA	BB	Total	R	*p* value
HMEC only CTCF BSs	7241	*1655*	947	4775	14,618	2.34	10^−168^
HUVEC only CTCF BSs	4516	264	*1180*	1524	7484		
Common CTCF BSs	24,986	2587	4647	9750	41,970		

(B) H3K9ac; GM12878-NHEK	AA	AB	BA	BB	Total	R	*p* value
GM12878 only H3K9ac peaks	14,695	*3111*	596	1708	20,110	4.54	< 10^−300^
NHEK only H3K9ac peaks	19,997	1401	*5949*	4154	31,501		
Common H3K9ac peaks	21,594	2036	1078	2911	27,619		

(C) H3K27me3; MCF7-GM12878	AA	AB	BA	BB	Total	R	*p* value
MCF7 only H3K27me3 peaks	5213	1751	*3637*	9712	20,313	0.54	10^−122^
GM12878 only H3K27me3 peaks	7176	*1765*	1145	3608	13,694		
Common H3K27me3 peaks	318	95	118	317	848		

Preference to cell-type specific A/B compartment is emphasized in italics

To study the relation between cell-type-specific binding sites and compartments across many TFs, we focused on GM12878 and K562, which have ChIP-seq data for 49 common TFs. Strong cell-type-specific TFBS-compartment relationship was observed for the vast majority of TFs (Fig. 3b). We obtained a significant relationship for 44 out of 49 TFs (FDR < 0.05). (Three out of the five TFs with nonsignificant *p* value have very small group sizes, and thus, their tests lack statistical power.) The strongest effect was observed for EP300, a transcriptional activator that marks active enhancers. In the analysis of other pairs of cell lines, we found a similar significant preference of cell-type-specific TF binding events for cell-type-specific A compartment (Additional file 2: Fig. S4). EZH2, a member of the polycomb-group (PcG) family that maintains suppressive chromatin state, was markedly different than other TFs and generally showed mild preference to cell-type-specific B compartments (Additional file 2: Fig. S4).

Next, we carried out similar tests for epigenetic marks that were profiled by ENCODE in cell lines for which we analyzed Hi–C data. In accordance with previous reports, most histone modifications showed a significant enrichment for the A compartment (Additional file 2: Fig. S5). Exceptional to this rule were H3K27me3, which is associated with polycomb repression, and H3K9me3, which is associated with heterochromatin. These two repressive marks were typically less enriched for A, and the latter showed a significant enrichment for the B compartment in HMEC and HUVEC cell lines (Additional file 2: Fig. S5). Based on pairwise comparisons between cell lines, we found that while most histone modifications show preference to cell-type-specific A compartments, H3K27me3 and H3K9me3 tend to show preference to cell-type-specific B compartments (Fig. 3c). Specific examples for H3K9ac, which marks transcriptionally active regions, and for H3K27me3 are given in Table 2b, c, respectively (and in Additional file 1: Table S4a, b). Despite the significant preference to cell-type-specific A or B compartments, a large portion of cell-type-specific histone modification events occur within AA or BB regions (Table 2b, c).

### Association between extent of promoter interactions and basal gene expression

**Fig. 4:**
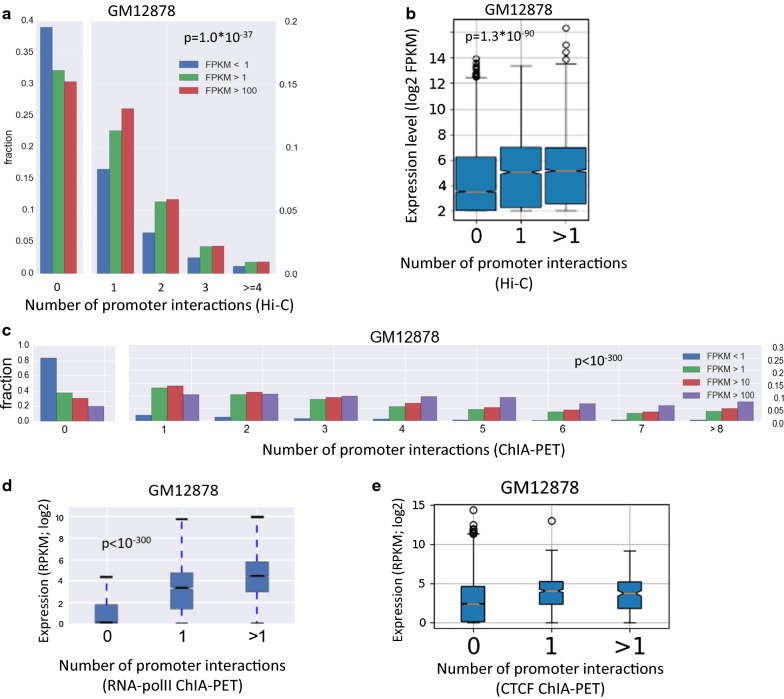
Gene expression levels versus promoter interactions in the A compartment. **a** Genes in compartment A were partitioned into three groups according to their expression levels. For each group, the distribution of genes over bins of number of promoter interactions, inferred from Hi–C data, is shown. *p* value was calculated using Wilcoxon’s test comparing the distributions in the least and most abundant expression groups. **b** Genes in compartment A were partitioned into three groups according to the number of interactions their promoters are engaged in, and the distributions of gene expression levels were compared (*p* value is for Wilcoxon’s test comparing the group of genes with 0 interactions to the genes with at least one interaction). **c**, **d** The same analysis as in B and A, respectively, but here using promoter interactions derived from RNA PolII ChIA-PET data in the GM12878 cell line. **e** The same analysis as in **d**, but using CTCF ChIA-PET data (in GM12878)

The A compartment is generally characterized by high transcriptional activity. Yet, genes within this compartment show considerable expression variability and many of them are not expressed at any detectable level. Our next analysis thus focused on genes within the A compartment and examined the relationship between the extent of chromatin interactions at promoter regions and gene expression level. In this analysis, we first used promoter–enhancer interactions inferred from Hi–C data by the PSYCHIC tool [16]. We expected that, per cell type, promoters of highly expressed genes would show stronger engagement in chromatin interactions than promoters of lowly expressed genes. Indeed, in all five cell lines that we tested, we found a significant positive association between the number of interactions in which a promoter is involved and the gene’s expression level (Fig. 4a, b; Additional file 2: Fig. S6A-B). We next applied a similar test, but this time using experimental promoter interactions derived from ChIA-PET data for RNA polymerase II in three cell lines (K562, GM12878 and MCF7). Here too, for all three cell lines examined, we found a highly significant positive association between extent of promoter interactions and gene expression level (Fig. 4c, d; Additional file 2: Fig. S6C, D). A caveat of this analysis using RNA PolII ChIA-PET is that PolII signal at promoters is correlated with expression level and this effect could confound the estimation of the number of chromatin interactions in which the promoter is involved. We repeated this analysis using CTCF ChIA-PET data in GM12878 cell line [17]. Interestingly, while promoters involved in chromatin interactions showed significantly higher expression than those that were not involved, promoters that were involved in multiple CTCF interactions had slightly lower expression than those involved in a single interaction (Fig. 4e), indicating that there is no simple relationship within the A compartment between the number of chromatin interactions a promoter is engaged with and its level of transcriptional activity.

**Fig. 5:**
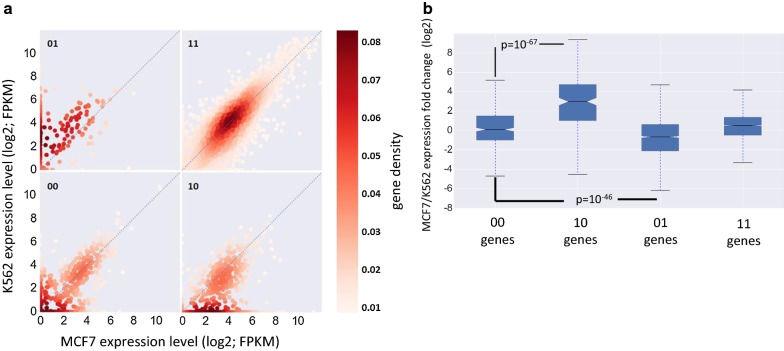
Differential promoter interactions versus differential gene expression. **a** Genes located in the A compartment in both MCF7 and K562 cell lines (AA genes) were divided into four groups according to the engagement of their promoters in chromatin interactions in the two cell lines (as indicated by RNA PolII ChIA-PET data). Gene sets 00, 01, 10, and 11 correspond, respectively, to the set of genes whose promoter is engaged in chromatin interactions in either none, only K562, only MCF7 or both cell lines. Expression levels in both cell lines are plotted for each gene set. Color indicates gene density. **b** Distribution of fold change in gene expression (log2) between MCF7 and K562 was calculated for each gene set. Highly significant association between differential gene expression and differential involvement of promoters in chromatin interaction was observed. *P* values are computed using Wilcoxon’s test

The above analyses were done on each cell line separately. We next examined correlation between dynamic promoter interactions and gene expression across cell lines. Specifically, we tested whether changes in a gene’s expression over different cell lines are associated with differences in the number of interactions involving the gene’s promoter in these cell lines. This analysis too was confined to genes located within the A compartment in both cell lines (“AA” genes). For each pair of cell lines, we divided the genes into four groups, based on RNA Pol-II ChIA-PET data: no promoter interactions in both cells (“00” group); promoter interactions detected only in cell line 1 (“10” genes); only in cell line 2 (“01” genes) and in both (“11” genes). Notably, differential gene expression between pairs of cell lines was strongly associated with differential engagement of promoters in chromatin interactions (Fig. 5a, b for MCF7 vs. K562). Similar results were obtained for the other pairs that we examined (data not shown). These results suggest that dynamic, intra-TAD chromatin interactions involving gene promoters within the A compartment modulate cell-type-specific gene expression.

### Association between basal chromatin organization and treatment-induced TF binding profiles

**Fig. 6:**
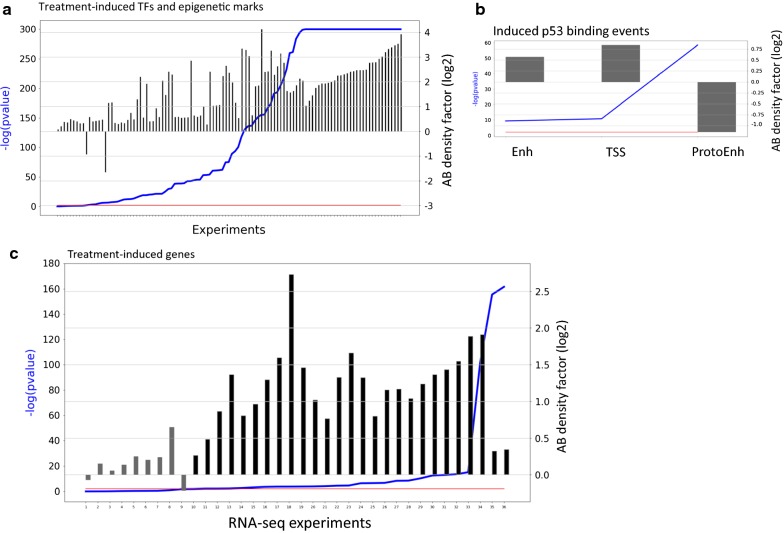
Enrichment of treatment-induced TFBSs and genes in the basal A compartment. **a** TFBSs induced by various treatments are significantly enriched in the A compartment determined in the cells under basal condition. Experiments are sorted by *p* value, and enrichment ratios are represented by bars. Red line: *p* value = 0.01. **b** Enrichment for A/B compartments of three classes (TSS, enhancer and protoenhancer) of p53 binding events induced by Nutlin-3a treatment in IMR90 cell lines. **c** The same as A, but for treatment-induced genes

Many transcriptomic studies demonstrated that a large portion of the transcriptional response to various challenges is cell-type specific [18–20]. Surprisingly, recent epigenomic and transcriptomic analysis of the response to TNF-α observed that enhancers activated by this trigger were already in contact with their target promoters before treatment [15]. Therefore, we next sought to examine the role of basal chromatin interactions, which are in place in cells before any challenge is applied, in shaping cell-type responses induced by treatment. To allow us to draw general conclusions, we analyzed a variety of cell lines and multiple treatments covering diverse biological processes. We first analyzed 110 publicly available ChIP-seq datasets, recorded in cells for which we analyzed Hi–C data, that profiled TF binding and epigenetic marks before and after the application of various treatments. Overall, we analyzed 21 TFs in seven cell lines in response to 22 treatments. Per experiment, we analyzed TFBSs detected under basal and stress conditions and identified the set of TFBSs that were induced in response to treatment. We then divided these induced TFBSs into A/B compartments. For the vast majority of experiments (> 90%), we observed a highly significant preference of the induced sites to the A compartment, suggesting that the preexisting A/B compartmentalization within a cell line constrains TF-chromatin interactions that are induced in response to stress (Fig. 6a and Additional file 1: Table S5).

One of the datasets that we analyzed profiled p53 binding sites after its activation by Nutlin-3a treatment in IMR90 cells [21]. This study divided the induced p53 binding events into three classes: promoter/TSS events (p53 peaks that overlapped H3K4me3 regions; 918 events), enhancer events (p53 peaks that overlapped H3K4me1 regions; 1558 events), and a third class called “protoenhancer”/”distal” events in which p53 peaks overlapped neither H3K4me3 nor H3K4me1 regions (1942 events). Intersection with ATAC-seq data showed that most distal p53 binding events occurred within inaccessible chromatin regions, indicating that p53 has the capacity to act as a pioneering factor, which can bind its response element in the context of a nucleosome. Here, we intersected these three classes of p53 binding events with A/B compartmentalization of (basal) IMR90 cells. Interestingly, while the TSS and enhancer classes were significantly enriched for the A compartment, the “protoenhancer” binding events were strongly enriched for the B compartment (Fig. 6b). Of note, based on GRO-seq and PolII ChIP-seq signals it was shown that contrary to p53 binding events in TSSs and enhancers, the “protoenhancer” events do not result in activation of these p53 binding sites or their engagement in active transcription (namely, apparently, these events do not affect gene expression) [21]. Thus, these results further suggest that the 3D organization of the genome in each cell type restrains the regulatory impact of TF binding events and is among the factors that determine the subsets of events that will (or will not) affect gene transcription.

Next, to examine the relationship between cell-type-specific chromatin organization and response to treatment more directly, we sought ChIP-seq datasets that profiled the same TF in response to the same treatment in different cell lines (for which we also analyzed Hi–C data). Several experiments that examined responses to TNF-α and estradiol met this requirement. For each pair of cell lines treated by the same agent and profiled for the same TF, we again divided the induced TFBSs into three groups: binding sites induced upon treatment only in cell line 1, binding sites induced only in cell line 2, and binding sites induced in both. Induced TFBSs in each group were then divided into four categories—AA, AB, BA, and BB as defined above. In all comparisons, TFBSs induced only in cell line 1 showed a significant preference for AB regions over the BA ones, and vice versa for TFBSs induced only in cell line 2 (Table 3; Additional file 1: Table S6). This result further demonstrates the strong relationship between cell-specific basal genome organization and the landscape of TF-chromatin interactions that are induced upon challenge. Yet, despite the significant association between cell-type-specific TF binding induction and chromatin organization, most of the cell-type-specific induced TFBSs were located in AA regions (Table 3; Additional file 1: Table S6), again indicating that factors other than A/B compartmentalization play more dominant role in determining cell-type-specific TF binding profiles.

**Table 3:** Cell-type-specific treatment-induced TFBSs show preference to cell-type-specific A compartment

MCF7–T47D; treatment: estradiol antibody: ER	AA	AB	BA	BB	Total	A/B enrichment	R	*p* value
MCF7 only ESR1 BSs	9656	*2356*	1459	3285	16,756	1.61	1.61	3.29E−29
T47D only ESR1 BSs	2031	257	*410*	475	3173	1.6		
Common ESR1 BSs	3138	406	330	461	4335			

Preference to cell-type specific A/B compartment is emphasized in italics

### Association between basal chromatin organization and transcriptional response to treatment

The above analyses examined the association between basal chromatin 3D organization and treatment-induced TF-chromatin interactions. Next, we examined the association between basal chromatin 3D organization and gene induction in response to treatment. For this goal, we analyzed 36 gene expression datasets that recorded transcriptome profiles in response to various challenges (in cell lines for which we have analyzed Hi–C data). For each cell line and treatment, we tested whether the set of genes that were induced upon treatment was over-represented in the A compartment (taking into account the general gene enrichment in this compartment). Indeed, in most conditions, we observed a significant preference of the induced genes to the A compartment (Fig. 6c; Additional file 2: Fig. S7 and Additional file 1: Table S7). This suggests that the preference of induced TFBSs to the pre-challenge A compartment leads to an induced transcriptional response that show similar preference. The statistical significance obtained by the analysis of the induced genes was usually lower than that obtained by the induced TFBSs since the numbers of responsive genes were substantially lower than the numbers of induced TFBSs. Nevertheless, 28 out of 34 experiments had a significant *p* value (FDR < 0.05) and 32 out of 34 experiments had enrichment factor larger than 1.0 (*p* < $3.5 \times 10^{ - 8} ;\,{\text{binomial test}}$).

**Fig. 7:**
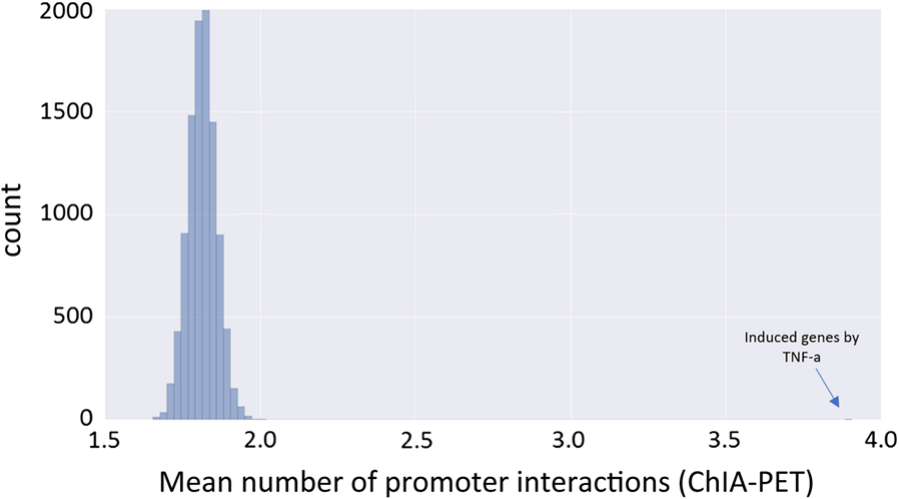
Engagement of promoters of treatment-induced genes in basal chromatin interactions. We used permutation tests to assess the significance of the engagement in chromatin interactions observed for promoters of genes that were induced upon challenges. The figure shows the analysis for the set of genes that were induced in GM12878 cells upon TNF-α treatment (the positive set). Ten thousand randomly selected gene sets of the same size and with the same basal expression distribution as the positive set were used to generate a null distribution. The mean number of promoter interactions per gene (3.9) was significantly higher for the positive set

In a previous section, we described an association between the extent of promoter interactions and basal gene expression level (Fig. 4). Here, we examined whether promoters of genes, within compartment A, that were induced in response to challenges also show higher involvement in chromatin interactions that already exist in the cells under basal condition. Analyzing numerous RNA-seq datasets, we systematically observed that promoters of induced genes were engaged, already in basal conditions, in a markedly higher number of chromatin interactions compared to promoters of noninduced genes that are located in the A compartment and have comparable basal expression levels. We estimated the significance of this higher degree of chromatin interaction by using a permutation test with 10,000 iterations, in each iteration selecting a random set of genes (from A compartment) of the same size as the induced genes set. Expression level was controlled for by dividing the A-compartment genes into 10 bins, according to their basal expression level, and generating random gene sets having the same distribution as the test set of the induced gene. In all experiments except one (with very low number of included genes and thus limited statistical power), we obtained significant *p* values (*p* < 0.05) (Fig. 7; Additional file 1: Table S8).

Last, we examined whether cell-type-specific gene induction in response to treatment correlates with pre-existing chromatin compartmentalization. We focused on response to TNF-α as we gathered RNA-seq datasets that profiled responses to this trigger in five different cell lines for which we determined AB compartmentalization based on Hi–C data (HMEC, IMR90, GM12878, MCF7 and HUVEC). We followed the same analysis that we applied above to TFBSs that were induced in a cell-type-specific manner (Table 3), and applied it to the set of TNF-α-induced genes. For 8 out of 10 pairwise comparisons we found a significant association: Genes induced specifically in cell line 1 were enriched for AB over BA regions (and vice versa for genes specifically induced in cell line 2) (Table 4; Additional file 1: Table S9). Notably, in this analysis too, the majority of cell-type-specific responsive genes were located in AA regions, again indicating that other factors play critical roles in determining the specific spectrum of genes that respond to a challenge in each cell type.

**Table 4:** Cell-type-specific treatment-induced genes show preference to cell-type-specific basal A compartment

HMEC–MCF7	AA	AB	BA	BB	Total	*R*	Enrichment	*p* value
Induced only in HMEC	29	*20*	6	9	64	3.44	2.0	0.00095
Induced only in MCF7	267	22	*36*	33	358	1.67		
Induced in both	34	4	1	4	43			

Preference to cell-type specific A/B compartment is emphasized in italics

## Discussion

To further explore links between the 3D organization of the genome and gene regulation, we have analyzed together Hi–C data from 13 different human cell lines and numerous ChIP-seq and RNA-seq experiments that were recorded in the same cell lines under basal conditions and in response to various treatments. We first confirmed the strong relationship between the partition of the genome into the A/B compartments and transcriptional activity. In all cell lines, expression level of genes located within the A compartment was significantly higher than expression level of genes located in B (Fig. 1b, c), and differential expression between cell lines correlated with differences in AB compartmentalization (Fig. 2). Similarly, in analysis of 122 TF ChIP-seq datasets, the binding profile of the vast majority of TFs showed a significant preference for the A compartment (Additional file 2: Fig. S3), and cell-type-specific TF binding events correlated with cell-type-specific A compartments (Fig. 3a, b). Most histone modifications that we examined showed similar tendency, except the repressive marks H3K27me3 and H3K9me3 that mostly showed the opposite preference (Fig. 3c). These correlative results suggest an important effect for the higher-order chromosomal organization on TF-chromatin interaction profiles. Yet, in comparisons of TF binding profiles between different cell lines, the majority of cell-type-specific TFBSs were located in genomic regions that are assigned to A compartment in both cell lines (AA regions) (Table 2). This observation indicates that other factors play stronger roles than A/B compartmentalization in shaping the landscape of TF-chromatin interactions in each cell type. Master TFs that establish and maintain cell identify are likely a major factor. These regulators exhibit highly cell-type-specific expression pattern and were shown to have great impact on the selection of TF binding sites in different cell types [22, 23].

Using E–P links derived from Hi–C and ChIA-PET data, we found, for genes located within the A compartment, a significant correlation between gene expression levels and the extent to which promoters are engaged in chromatin interactions. This association was most pronounced in the comparison between genes with no interaction at all and those that have at least one (Fig. 4). Moreover, we showed that differential expression between cell types is associated with dynamic change in involvement of promoters in such interactions (Fig. 5), which are most likely mediated by cell-type-specific TFs. A recent study showed that during cell reprogramming, the expression of lineage-specific TFs drives genome reorganization that precedes changes in gene expression [24].

We then turned to analyze the association between the organization of the genome under basal conditions and transcriptional programs that are induced in response to various triggers. First, we showed that, typically, both induced TF binding events and induced genes are enriched in the A compartment (Fig. 6a, c), suggesting that preexisting A/B compartments within a cell constrain its network of induced TF-chromatin interactions and activated genes. We then demonstrated an association between cell-type-specific response to triggers and basal cell-type-specific AB compartmentalization. Cell-type-specific induced TF binding and activated genes show a significant enrichment for cell-type-specific A compartments (Tables 3, 4). Yet, here too, a large portion of the cell-type-specific induced TFBS and genes are located in genomic regions that are assigned to the A compartment in both responsive and nonresponsive cell lines, further underscoring that additional key factors participate in shaping the specific transcriptional response to challenges elicited in each cell type. A limitation of the analysis that we performed is the lack of Hi–C data in the treated cells. We therefore do not have direct data on the treatment-induced changes in A/B compartmentalization. Future experiments that will examine this aspect could greatly improve our understanding of the interplay between the dynamic modulation of the genome organization and gene expression programs.

Current techniques for determining the 3D organization of the genome are still limited in their resolution and sensitivity. Further development of these methods together with advances in their application to single cells will allow us to better understand how the genome organization in different cells is causally linked to cell-type-specific transcriptional programs both under basal conditions and in response to various challenges.

## Conclusions

Collectively, the large-scale meta-analysis that we carried out in this study further demonstrates the strong association between cell-type-specific A/B compartmentalization, modulation of landscape of TF-chromatin interactions, and differential gene expression. Moreover, our results further suggest a role for the 3D organization of the genome under basal conditions, at the layers of both A/B compartmentalization and intra-TAD enhancer–promoter interactions, in shaping TF binding events and the network of genes that are induced in response to treatment. Yet, our pairwise comparisons also show that most events of differential TF binding and gene induction occur in genomic loci assigned to A compartment in both cell types, underscoring the role of additional critical factors in determining transcriptional programs that are active in each cell type.

## Methods

### Identification of A and B compartments from Hi–C data

We defined A/B compartments for 13 human cell lines for which Hi–C data are available (Additional file 1: Table S1). Identification of A and B compartments was performed similarly to what has been previously described [5, 11]. Briefly, Hi–C contact frequency matrix was first normalized using the Knight and Ruiz matrix balancing method [25]. Then, we performed principal component analysis (PCA) for each intrachromosomal matrix separately at 100-Kb resolution. In most cases, the first principal component vector partitions the chromosome into two compartments, A and B, according to the sign of the elements. In other cases, mostly in short chromosomes, the first principal component divides the chromosome to its two arms and the second component partitions it to the A/B compartments. As seen in previous studies [7], the A compartment is gene rich and its chromatin is less dense, while the B regions are gene poor and their chromatin is denser. Thus, we determined, for each chromosome separately, whether positive or negative values of the PC that indicates the A/B compartmentalization correspond to A or B based on gene richness; the compartment with higher gene density was labeled as A compartment. Centromeric regions were not included in the A/B partitions since no chromatin interactions are identified by Hi–C in these regions.

### RNA-seq analysis

RNA-seq data were analyzed using a standard pipeline. Briefly, raw sequence data were downloaded from GEO/SRA DB and mapped to the human genome (hg19) using TopHat2 [26]. The number of reads that mapped to each annotated gene was counted using HTSeq-counts [27] based on GENCODE annotations [28]. Gene expression estimates were normalized to RPKM. In the comparison of expression profiles between treated and control samples, we defined the genes whose expression was changed by at least 1.5-fold as differential ones (to avoid inflation of lowly expressed genes among the called differential genes we used a floor level of 1.0 RPKM). In addition, for datasets that included replicates, we used DESeq 2 [29] to define the set of differential genes (using FDR of 5%).

### ChIP-seq analysis

To ensure analysis uniformity, we did not rely on peaks called by original studies, but downloaded raw sequence data and detected TF peaks ourselves. Briefly, for each ChIP-seq experiment, reads were aligned to the human genome (hg19) using Bowtie2 [30] and peaks were called using MACS2 by comparing IP and input samples. For detection of peaks induced upon treatment, IP samples measured under control and treated conditions were directly compared [31].

### AB density factor *D*

For each transcription factor and cell line we computed the *AB density factor*, *D*, defined as follows: Let the number of observed binding sites in region *S* be *O*(*S*) and number of expected binding sites in region *S* be *E*(*S*):

$$D = \frac{O\left( A \right)/O\left( B \right)}{E\left( A \right)/E\left( B \right)}$$

*D* > 1 implies that binding sites are enriched for A compartment, and *D* < 1 implies that binding sites are enriched for B compartment. For TF binding sites, *E*(*A*)/*E*(*B*) is equal to the ratio between the genomic size of the two compartments. For induced genes, *E*(*A*)/*E*(*B*) is equal to the ratio between the number of genes located within these two compartments.

### AB occupancy enrichment ratio R

For pairwise comparisons between cells, to test whether cell-type-specific TFBSs occur more often in regions assigned as A compartment in the cell line where the binding event was detected and as B in the other cell line than the opposite assignment, we defined the *AB occupancy enrichment ratio* R, as follows: Let the number of BSs in region S occurring only in cell line *i* be $n\left( {i,S} \right)$. Then

$$R = \frac{{n\left( {1,AB} \right) + n\left( {2,BA} \right)}}{{n\left( {1,BA} \right) + n\left( {2,AB} \right)}}.$$

## Additional files

**Additional file 1: Table S1.** Hi-C datasets. **Table S2.** Encode ChIP-seq data included in our analyses (122 TFs profiled in cell lines with Hi-C data). **Table S3.** Enrichment of CTCF binding sites for the A compartmentalization. **Table S4A.** Enrichment of cell-type-specific H3K9ac events for cell-type-specific A compartment over B compartment. **Table S4B.** Enrichment of cell-type-specific H3K27me3 events for cell-type-specific B compartment over A compartment. **Table S5.** Preference of induced TF binding sites and epigenetic marks to the A compartment. **Table S6.** Binding site induction and compartmentalization in two cell lines under the same treatment, for a particular TF. **Table S7.** Preference of induced genes to the A compartment. **Table S8.** Promoters of induced genes are involved, in basal condition, in higher numbers of chromatin interactions. **Table S9**. Preference of cell-type-specific induced genes to cell-type-specific A compartment.

**Additional file 2: Fig. S1.** Correlation between the magnitude of the PC that represents A/B compartmentalization and gene expression level. **Fig. S2.** Association between changes in A/B compartmentalization and differential gene expression between cell types. A. For each pair of cell lines, we examined the difference (fold change) in expression level between genes assigned to the AB and BA sets (for a pair of cell lines 1 and 2, AB: genes located in the A compartments in cell line 1 and in B in cell line 2; BA: genes located in the B compartment in cell line 1 and in A in cell line 2). For 27 out of 28 pairwise comparisons (all except HMEC–NHEK), we observed a highly significant association (FDR ≪ 5%) between differential compartmentalization and expression. (*p* values calculated using Wilcoxon’s test.) B. Correlation between the change in the magnitude of PC1 and change in gene expression level in the comparison between GM12878 and four other cell lines. **Fig. S3**. Enrichment of TFBSs in the A compartment. ChIP-seq experiments are sorted by *p* value, and A-B density factors are represented by bars. Red line indicates *p* value = 0.01. Shown are experiments in the GM12878 cell line. Similar results were observed for all other cell lines (data not shown). **Fig. S4**. Preference of cell-type-specific TF binding for cell-type-specific compartments. Preference of cell-type-specific TF binding events to AB genomic regions over BA regions is measured by the *AB occupancy enrichment ratio*. The compared cell lines and the examined TF are indicated below each bar (cell lines are indicated by the first two letters of their name, e.g., GM = GM12878, HU = HUVEC, K5 = K562). **Fig. S5**. Enrichment of histone modifications in the A/B compartments. The analysis presented in Fig. S3 for TF binding events is applied here to histone modification peaks. Most modifications showed a significant enrichment for the A compartment. The repressive marks H3K27me3 and H3K9me3 showed markedly lower enrichment, and the latter was even significantly enriched in the B compartment (in HMEC and HUVEC cell lines). **Fig. S6**. Gene expression levels vs. promoter interactions in compartment A. The same analyses described in the legend of Fig. 4A-D are applied here to additional cell lines. **Fig. S7**. Enrichment of treatment-induced genes in the basal A compartment. The same analysis as in Fig. 6C, but calling differential genes based on FDR of 5% (rather than based on fold change criterion).
