# Supplementary material for: Genomic meta-analysis of the interplay between 3D chromatin organization and gene expression programs under basal and stress conditions
Source: Epigenetics Chromatin. 2018 Aug 29;11:49. doi: 10.1186/s13072-018-0220-2 (PMC6114837; doi:10.1186/s13072-018-0220-2)
Supplement: Supplementary file 1 — Additional file 1: Table S1. Hi-C datasets. Table S2. Encode ChIP-seq data included in our analyses (122 TFs profiled in cell lines with Hi-C data). Table S3. Enrichment of CTCF binding sites for the A compartmentalization. Table S4A. Enrichment of cell-type-specific H3K9ac events for cell-type-specific A compartment over B compartment. Table S4B. Enrichment of cell-type-specific H3K27me3 events for cell-type-specific B compartment over A compartment. Table S5. Preference of induced TF binding sites and epigenetic marks to the A compartment. Table S6. Binding site induction and compartmentalization in two cell lines under the same treatment, for a particular TF. Table S7. Preference of induced genes to the A compartment. Table S8. Promoters of induced genes are involved, in basal condition, in higher numbers of chromatin interactions. Table S9. Preference of cell-type-specific induced genes to cell-type-specific A compartment. [file 13072_2018_220_MOESM1_ESM.pdf]

Table S1. Hi-C datasets

Cell line	Description	Hi-C data resolution (Kbp)	Source study
**MCF10A**	Non-tumorigenic epithelial breast cell line	40, 250	[1]
**MCF7**	Breast cancer cell line with overexpression of estrogen receptor		
**LNCAP**	Androgen sensitive prostate adenocarcinoma cell line	40, 100	[2]
**PrEC**	Prostate epithelial cell line	40	
**PC3**	Androgen insensitive prostate cancer cell line		
**HUVEC**	Human umbilical vein endothelial cell line	5, 10, 100, 250, 500, 1000	[3]
**NHEK**	Primary normal human epidermal keratinocytes cell line		
**IMR90**	Fetal lung fibroblasts cell line		
**K562**	Myelogenous leukemia cell line		
**KBM7**	Chronic myelogenous leukemia cell line		
**HMEC**	Human mammary epithelial cell line		
**GM12878**	Lymphoblastoid cell line	1, 5, 10, 100, 250, 500, 1000	
**T47D**	Breast cancer cell line	100	[4]

Table S2. ENCODE ChIP-seq data included in our analyses (122 TFs profiled in cell lines with Hi-C data)

Transcription factor	Cell lines with available ChIP-Seq dataset in ENCODE
ZBTB33	K562, GM12878
CTCF	K562, HUVEC, NHEK, HMEC, IMR90, GM12878
EGR1	K562, GM12878
RUNX3	GM12878
MAZ	K562, GM12878
RAD21	K562, GM12878, IMR90
SMC3	K562, GM12878
MAFK	K562, IMR90
MAFF	K562
E2F6	K562
MAX	K562, GM12878, HUVEC
PAX5	GM12878
POLR2A	K562, HUVEC, GM12878, IMR90, NHEK
PHF8	K562
PML	K562, GM12878
YY1	K562, GM12878
TAF1	K562, GM12878
SIN3AK20	K562
GTF2F1	K562
ATF2	GM12878
MYC	K562, GM12878, HUVEC
MXI1	GM12878, K562
JUND	K562, GM12878
POU2F2	GM12878
KDM5B	K562
TBP	K562, GM12878
EP300	K562, GM12878

ELK1	GM12878, K562
RFX5	K562, GM12878
CHD2	K562, GM12878
ATF3	K562, GM12878
BRCA1	GM12878
NFYA	K562, GM12878
NFYB	K562, GM12878
JUN	K562, HUVEC
GABPA	K562, GM12878
E2F4	K562, GM12878
SP1	K562, GM12878
SRF	K562, GM12878
ELF1	K562, GM12878
USF1	K562, GM12878
ATF1	K562
SIX5	K562, GM12878
USF2	GM12878, K562
FOS	K562, HUVEC, GM12878
TBL1XR1	K562, GM12878
ZNF143	K562, GM12878
SP2	K562
EBF1	GM12878
CTCFL	K562
TEAD4	K562
THAP1	K562
ZEB1	GM12878
CEBPB	K562, IMR90, GM12878
PBX3	GM12878
UBTF	K562
CBX3	K562
BCLAF1	K562, GM12878

RBBP5	K562
RCOR1	K562, GM12878
FOSL1	K562
GATA2	K562, HUVEC
BHLHE40	K562, GM12878
TAL1	K562
BCL3	GM12878, K562
NFATC1	GM12878
MEF2A	GM12878, K562
MEF2C	GM12878
ZNF263	K562
CCNT2	K562
HDAC2	K562
TCF3	GM12878
TCF12	GM12878
ZNF274	K562, GM12878
STAT1	GM12878
BATF	GM12878
HMGN3	K562
SETDB1	K562
TAF7	K562
SPI1	K562, GM12878
ETS1	K562, GM12878
REST	K562, GM12878
ZBTB7A	K562
EZH2	NHEK, HMEC, K562, HUVEC, GM12878
JUNB	K562
NR2F2	K562
TRIM28	K562
GTF3C2	K562
SAP30	K562

CHD1	K562, GM12878
STAT5A	K562, GM12878
HDAC1	K562
NRF1	K562, GM12878
NR2C2	GM12878, K562
SIN3A	GM12878
GATA1	K562
NFIC	GM12878
IRF4	GM12878
BCL11A	GM12878
MTA3	GM12878
FOXM1	GM12878
RXRA	GM12878
KAP1	K562
BACH1	K562
HDAC8	K562
NFE2	K562, GM12878
ARID3A	K562
WRNIP1	GM12878
GTF2B	K562
HDAC6	K562
SMARCA4	K562
BRF2	K562
IKZF1	GM12878
SMARCB1	K562
STAT3	GM12878
BDP1	K562
RPC155	K562
SIRT6	K562
RDBP	K562
ZZZ3	GM12878

POLR3G	K562, GM12878
BRF1	K562

Table S3. Enrichment of CTCF binding sites for the A compartmentalization.

Cell line	Observed A	Observed B	total	Expected A	Expected B	D	-log 10 p-value
**K562**	48,141	15,524	63,665	32,056	31,599	3.06	> 300
**HUVEC**	35,329	12,544	47,873	24,216	23,656	2.75	> 300
**NHEK**	45,665	16,701	62,366	32,700	29,665	2.48	> 300
**HMEC**	36,469	18,135	54,604	26,322	28,281	2.16	> 300
**IMR90**	29,184	14,880	44,064	21,121	22,942	2.13	> 300
**GM12878**	42,295	17,575	59,870	28,970	30,899	2.57	> 300

Table S4A. Enrichment of cell-type specific H3K9ac events for cell-type specific A compartment over B compartment

	AA	AB	BA	BB	total	R	-log10 p-value
**HMEC vs. GM12878**							
**BSs only in Cell1**	17789	**6309**	1708	5087	30893	4.48	> 300
**BSs only in Cell2**	15292	567	**3880**	1779	21518		
**Common BSs**	21558	1201	2319	3613	28691		
**HUVEC vs. GM12878**							
**BSs only in Cell1**	16635	**6747**	651	3805	27838	7.64	> 300
**BSs only in Cell2**	16046	612	**2900**	1844	21402		
**Common BSs**	20606	1173	966	2614	25359		
**HUVEC vs. HMEC**							
**BSs only in Cell1**	14992	**4292**	468	3168	22920	4.16	> 300
**BSs only in Cell2**	16370	1369	**3341**	4301	25381		
**Common BSs**	27220	2629	984	4031	34864		
**K562 vs. GM12878**							
**BSs only in Cell1**	21793	**4294**	1019	3916	31022	4.56	> 300
**BSs only in Cell2**	14158	580	**2994**	1849	19581		
**Common BSs**	24463	1239	1350	2762	29814		
**K562 vs. HMEC**							
**BSs only in Cell1**	21915	**5083**	982	3672	31652	4.15	> 300
**BSs only in Cell2**	17015	1667	**5907**	5110	29699		
**Common BSs**	23268	2498	1285	3435	30486		
**K562 vs. HUVEC**							
**BSs only in Cell1**	22645	**3551**	1140	3770	31106	5.37	> 300
**BSs only in Cell2**	15827	627	**5944**	3772	26170		
**Common BSs**	22627	1193	1300	2594	27714		
**NHEK vs. GM12878**							
**BSs only in Cell1**	19997	**5949**	1401	4154	31501	4.54	> 300
**BSs only in Cell2**	14695	596	**3111**	1708	20110		
**Common BSs**	21362	1192	2042	3023	27619		
**NHEK vs. HMEC**							
**BSs only in Cell1**	11680	**1082**	471	2402	15635	1.52	26
**BSs only in Cell2**	7559	688	*678*	2879	11804		
**Common BSs**	42741	2511	2267	7437	54956		
**NHEK vs. HUVEC**							
**BSs only in Cell1**	18223	**3364**	1152	3363	26102	3.91	> 300
**BSs only in Cell2**	14273	584	**3426**	3056	21339		
**Common BSs**	26132	1241	2646	3560	33579		
**NHEK vs. K562**							
**BSs only in Cell1**	18782	**5447**	1599	3903	29731	3.46	> 300
**BSs only in Cell2**	21127	1132	**3998**	3561	29818		
**Common BSs**	23818	1357	2117	3026	30318		

Table S4B. Enrichment of cell-type specific H3K27me3 events for cell-type specific B compartment over A compartment

	AA	AB	BA	BB	total	R	p-value
**GM12878 vs. NHEK**							
**BSs only in Cell1**	7244	1099	**2136**	3306	13785	0.73	1.16E-18
**BSs only in Cell2**	4369	*1026*	1215	3272	9882		
**Common BSs**	350	71	62	288	771		
**GM12878 vs. K562**							
**MCF7 NHEK**							
**BSs only in Cell1**	5682	1460	**4114**	9381	20637	0.45	8.56E-113
**BSs only in Cell2**	4902	**1066**	904	3355	10227		
**Common BSs**	129	64	55	168	416		
**MCF7 vs. GM12878**							
**BSs only in Cell1**	5213	1751	**3637**	9712	20313	0.54	2.04E-122
**BSs only in Cell2**	7176	**1765**	1145	3608	13694		
**Common BSs**	318	95	118	317	848		

Table S5. Preference of induced TF binding sites and epigenetic marks to the A compartment

Cell line	treatment	antibody	induced in A	induced in B	log p-value	source
IMR90	TNF-a (10ng/mL) 1hr	p300	312	113	-25.05	[5]
IMR90	TNF-a (10ng/mL) 1hr	H3K4me3	487	104	-62.27	
IMR90	TNF-a (10ng/mL) 1hr	H3K36me3 (Abcam ab9050)	170	45	-19.06	
IMR90	TNF-a (10ng/mL) 1hr	PolII (Santa Cruz sc-899)	7613	1837	-300	
IMR90	TNF-a (10ng/mL) 1hr	flavopiridol (1ÂµM, 1hr)	9154	1655	-300	
HUVEC	TNF-a (10ng/mL) 1hr	H3K27ac (Abcam, ab4729)	5711	490	-300	
LNCAP	DHT (100nM, 2h) + TNF-alpha (1000U/ml, 2h)	anti-AR	4380	2816	-45.48	[6]
LNCAP	DHT (100nM, 2h) + TNF-alpha (1000U/ml, 2h)	anti-AR	4277	2751	-44.34	
LNCAP	DHT (100nM, 2h) + TNF-alpha (1000U/ml, 2h)	anti-AR	4611	3084	-38.47	
LNCAP	DHT (100nM, 2h) + TNF-alpha (1000U/ml, 2h)	anti-AR	4437	2943	-38.89	
LNCAP	DHT (100nM, 2h)	anti-AR	5301	3527	-45.45	
LNCAP	DHT (100nM, 2h)	anti-AR	5223	3550	-39.4	
LNCAP	DHT (100nM, 2h)	anti-AR	5475	3698	-42.89	
LNCAP	DHT (100nM, 2h)	anti-AR	5356	3662	-38.9	

LNCAP	DHT (100nM, 2h) + TNF-alpha (1000U/ml, 2h)	anti-FOXA1	4197	3270	-10.03	
LNCAP	DHT (100nM, 2h) + TNF-alpha (1000U/ml, 2h)	anti-FOXA1	3623	2858	-7.52	
LNCAP	DHT (100nM, 2h) + TNF-alpha (1000U/ml, 2h)	anti-FOXA1	6278	4755	-19.99	
LNCAP	DHT (100nM, 2h) + TNF-alpha (1000U/ml, 2h)	anti-FOXA1	6011	4524	-20.43	
LNCAP	DHT (100nM, 2h)	anti-FOXA1	181	157	-0.16	
LNCAP	DHT (100nM, 2h)	anti-FOXA1	146	138	-0.14	
LNCAP	DHT (100nM, 2h)	anti-FOXA1	6789	5540	-8.14	
LNCAP	DHT (100nM, 2h)	anti-FOXA1	6760	5385	-11.76	
LNCAP	TNF-alpha (1000 U/ml, 2h)	anti-FOXA1	222	168	-1.1	
LNCAP	TNF-alpha (1000 U/ml, 2h)	anti-FOXA1	189	147	-0.78	
LNCAP	TNF-alpha (1000 U/ml, 2h)	anti-FOXA1	506	404	-1.23	
LNCAP	TNF-alpha (1000 U/ml, 2h)	anti-FOXA1	502	402	-1.18	
LNCAP	DHT (100nM, 2h) + TNF-alpha (1000U/ml, 2h)	anti-PIAS3+PIAS1+PIAS2	2444	1751	-13.24	
LNCAP	DHT (100nM, 2h) + TNF-alpha (1000U/ml, 2h)	anti-PIAS3+PIAS1+PIAS2	2419	1749	-12.23	
LNCAP	DHT (100nM, 2h) + TNF-alpha (1000U/ml, 2h)	anti-PIAS3+PIAS1+PIAS2	832	622	-3.54	
LNCAP	DHT (100nM, 2h) + TNF-alpha (1000U/ml, 2h)	anti-PIAS3+PIAS1+PIAS2	843	637	-3.27	

LNCAP	DHT (100nM, 2h)	anti-PIAS3+PIAS1+PIAS2	1061	763	-5.96	
LNCAP	DHT (100nM, 2h)	anti-PIAS3+PIAS1+PIAS2	1022	751	-4.89	
LNCAP	DHT (100nM, 2h)	anti-PIAS3+PIAS1+PIAS2	2556	1713	-21.48	
LNCAP	DHT (100nM, 2h)	anti-PIAS3+PIAS1+PIAS2	2478	1686	-19.05	
LNCAP	TNF-alpha (1000 U/ml, 2h)	anti-PIAS3+PIAS1+PIAS2	97	69	-0.89	
LNCAP	TNF-alpha (1000 U/ml, 2h)	anti-PIAS3+PIAS1+PIAS2	86	66	-0.5	
LNCAP	TNF-alpha (1000 U/ml, 2h)	anti-PIAS3+PIAS1+PIAS2	880	460	-21.4	
LNCAP	TNF-alpha (1000 U/ml, 2h)	anti-PIAS3+PIAS1+PIAS2	810	485	-12.41	
LNCAP	TNF-alpha (1000 U/ml, 2h)	anti-p65	21	6	-1.93	
LNCAP	TNF-alpha (1000 U/ml, 2h)	anti-p65	21	7	-1.66	
LNCAP	TNF-alpha (1000 U/ml, 2h)	anti-p65	180	80	-7.14	
LNCAP	TNF-alpha (1000 U/ml, 2h)	anti-p65	168	76	-6.47	
LNCAP	DHT (100nM, 2h) + TNF-alpha (1000U/ml, 2h)	anti-p65	2075	1004	-61.01	
LNCAP	DHT (100nM, 2h) + TNF-alpha (1000U/ml, 2h)	anti-p65	2015	961	-61.49	
LNCAP	DHT (100nM, 2h) + TNF-alpha (1000U/ml, 2h)	anti-p65	2127	1041	-60.68	

LNCAP	DHT (100nM, 2h) + TNF-alpha (1000U/ml, 2h)	anti-p65	2008	1011	-53.12	
HUVEC	TNF-alpha (10 ng/ml, 30min)	anti-Pol3	867	50	-155.12	GSE34500
HUVEC	TNF-alpha (10 ng/ml, 30min)	anti-p65	15779	1040	-300	
IMR90	DMSO	H3K4me3 ChIP	15429	4242	-300	
IMR90	Nutlin-3a	H3K4me3 ChIP	14800	3896	-300	
IMR90	DMSO	H3K4me1 ChIP	13573	2528	-300	
IMR90	Nutlin-3a	H3K4me1 ChIP	1967	286	-300	
IMR90	DMSO	H3K27ac ChIP	15455	3236	-300	
IMR90	Nutlin-3a	H3K27ac ChIP	13167	2666	-300	
IMR90	DMSO	H4K16ac ChIP	3098	550	-300	
IMR90	Nutlin-3a	H4K16ac ChIP	1303	211	-192.97	
IMR90	DMSO	RNAPII ChIP	13161	2757	-300	
IMR90	Nutlin-3a	RNAPII ChIP	9070	1713	-300	
IMR90	DMSO	p53 ChIP	97	65	-2.62	
IMR90	Nutlin-3a	p53 ChIP	1637	734	-93.36	
IMR90	DMSO	H3K4me2 ChIP	27136	7113	-300	
IMR90	Nutlin-3a	H3K4me2 ChIP	25274	6516	-300	
MCF7	E2 for 45m	ERa	20417	8637	-300	[7]
MCF7	E2 for 45m	ERa	4268	1069	-300	
MCF7	IL1b for 45m	ERa	2813	911	-232.08	
MCF7	IL1b for 45m	ERa	62	7	-10.75	
MCF7	TNFa for 45m	ERa	5196	1538	-300	
MCF7	TNFa for 45m	ERa	45	15	-4.19	
MCF7	IKK7	ERa	1280	240	-166.56	
MCF7	IL1b+IKK7	ERa	19	6	-2.06	
MCF7	IKK7	ERa	57	42	-1.07	
MCF7	IL1b+IKK7	ERa	24	46	-1.78	
MCF7	E2+ICI	ERa	3739	540	-300	
MCF7	IL1b+ICI	ERa	1564	435	-151.48	
MCF7	E2+ICI	ERa	4	2	-0.36	
MCF7	IL1b+ICI	ERa	3328	1129	-259.85	
MCF7	E2+ICI	ERa	587	114	-75.34	
MCF7	IL1b+ICI	ERa	4520	819	-300	
MCF7	IL1b+ICI	ERa	1203	228	-155.61	
MCF7	E2 for 45m	p65	190	46	-21.49	
MCF7	E2 for 45m	p65	2	0	-0.53	
MCF7	IL1b for 45m	p65	244	50	-30.78	
MCF7	IL1b for 45m	p65	1560	322	-190.96	
MCF7	TNFa for 45m	p65	1534	437	-145.27	

MCF7	TNFa for 45m	p65	3175	1029	-261.56	
MCF7	Dexamethasone	GR E-20X sc-1003 Santa Cruz	8474	4131	-300	[8]
MCF7	Dexamethasone	GR E-20X sc-1003 Santa Cruz	6377	4410	-100.34	
MCF7	17ÃŸ-estradiol	ER cocktail: Ab-10 Thermo Scientific Lab Vision, HC-20 sc-543 Santa Cruz	4375	861	-300	
MCF7	17ÃŸ-estradiol	ER cocktail: Ab-10 Thermo Scientific Lab Vision, HC-20 sc-543 Santa Cruz	11153	4080	-300	
MCF7	Dexamethasone	FoxA1	6315	4018	-136.68	
MCF7	Dexamethasone	FoxA1	238	97	-15.29	
MCF7	17ÃŸ-estradiol	FoxA1	9178	7561	-53.4	
MCF7	17ÃŸ-estradiol	FoxA1	845	215	-89.05	
T47D	Dexamethasone	GR E-20X sc-1003 Santa Cruz	570	91	-74.81	
T47D	Dexamethasone	GR E-20X sc-1003 Santa Cruz	451	85	-54.06	
T47D	17ÃŸ-estradiol	ER cocktail: Ab-10 Thermo Scientific Lab Vision, HC-20 sc-543 Santa Cruz	2854	654	-293.27	
T47D	17ÃŸ-estradiol	ER cocktail: Ab-10 Thermo Scientific Lab Vision, HC-20 sc-543 Santa Cruz	3052	741	-299	
T47D	Dexamethasone	FoxA1	244	46	-29.61	
T47D	Dexamethasone	FoxA1	156	34	-17.44	
T47D	17ÃŸ-estradiol	FoxA1	303	42	-42.94	
T47D	17ÃŸ-estradiol	FoxA1	3266	903	-283.95	
T47D	Dexamethasone and 17ÃŸ-estradiol	FoxA1	12949	2332	-300	
K562	IFNa30	pol2	739	73	-117.62	ENCODE
K562	IFNa6h	pol2	838	86	-131.65	
K562	IFNg30	pol2	1342	152	-203.26	
K562	IFNg6h	pol2	1113	116	-173.65	
K562	IFNa30	cjun	2788	409	-300	
K562	IFNa6h	cjun	922	112	-136.38	
K562	IFNg30	cjun	2518	331	-300	
K562	IFNg6h	cjun	1583	232	-215.33	
K562	IFNa30	cmyc	3188	287	-300	
K562	IFNa6h	cmyc	5150	489	-300	
K562	IFNg30	cmyc	21370	2329	-300	

K562	IFNg6h	cmyc	11871	1181	-300	
GM1287 8	TNF	NFKB	4952	610	-300	

Table S6. Binding site induction and compartmentalization in two cell lines under the same treatment, for a particular TF.

	AA	AB	BA	BB	total	A/B Enrichment	R	p-value
**MCF7 - T47D; Treatment: Estradiol Antibody: ER** [8]								
Replicate 1								
**Cell1_only_BSs**	2354	522	230	534	3640	2.33	1.99	7.06E-23
**Cell2_only_BSs**	1177	149	233	295	1854	1.63		
**Common BSs**	1344	158	103	144	1749			
Replicate 2								
**Cell1_only_BSs**	7302	1834	1229	2751	13116	1.56	1.5	6.41E-13
**Cell2_only_BSs**	854	108	177	180	1319	1.63		
**Common BSs**	1794	248	227	317	2586			
Two rep combined								
**Cell1_only_BSs**	9656	2356	1459	3285	16756	1.61	1.61	3.29E-29
**Cell2_only_BSs**	2031	257	410	475	3173	1.6		
**Common BSs**	3138	406	330	461	4335			
**LNCAP - MCF7; Treatment: TNFa; Antibody: p65** [6]								
**Cell1_only_BSs**	74	28	16	43	161	1.7	1.59	0.0014
**Cell2_only_BSs**	1194	166	262	271	1893	1.56		
**Common BSs**	67	11	12	12	102			
**HUVEC - MCF7; Treatment: TNFa; Antibody: p65.** Genome-wide maps of RNA Polymerase II and p65 localization in HUVECs stimulated with TNF alpha (GSE34500)								
**Cell1_only_BSs**	12443	2568	321	842	16174	8	6.47	4.70E-99
**Cell2_only_BSs**	671	100	154	227	1152	1.44		
**Common BSs**	690	83	34	54	861			
**LNCAP – HUVEC; Treatment: TNFa; Antibody: p65** [6]								
**Cell1_only_BSs**	75	39	28	39	181	1.47	8.73	1.62E-37
**Cell2_only_BSs**	12237	374	3470	866	16947	10		
**Common BSs**	60	7	12	4	83			

Table S7. Preference of induced genes to the A compartment

Cell line	treatment	induced in A	induced in B	Log p-value	A/B enrichment	source
GM12878	TNF-a	3866	267	-101.57	3.76	ENCODE
IMR90	TNF-a (10ng/mL) 1hr	439	103	-6.43	1.74	[5]
IMR90	TNF-a (10ng/mL) 1hr	105	23	-2.12	1.82	
IMR90	cycloheximide (5Âµg/mL) pretreat 30min	254	46	-6.61	2.23	
IMR90	TNF-a (10ng/mL) 1hr; cycloheximide (5Âµg/mL) pretreat 30min	389	77	-8.39	2.06	
IMR90	TNF-a (10ng/mL) 1hr	80	27	-0.37	1.19	
HUVEC	IFN-G (50ng/mL) 2hr	124	10	-3.65	3.02	
MCF7	Estradiol (100nM) 160min	121	13	-3.73	2.75	
IMR90	Nutlin-3a	1771	558	-161.62	1.27	
MCF7	E2 for 3h	131	16	-3.49	2.45	[7]
MCF7	E2 for 3h	129	12	-4.51	3.16	
MCF7	IL1b for 3h	166	20	-4.4	2.51	
MCF7	IL1b for 3h	172	27	-3.07	1.95	
MCF7	IL1b+ICI for 3h	204	31	-3.79	2.02	
MCF7	IL1b+ICI for 3h	182	32	-2.56	1.75	
MCF7	TNFa for 3h	338	62	-3.99	1.7	
MCF7	TNFa for 3h	348	78	-2.12	1.4	
MCF7	TNFa+ICI for 3h	895	235	-1.82	1.2	
MCF7	TNFa+ICI for 3h	1045	382	-1.76	0.86	
MCF7	E2	252	31	-6.31	2.5	
MCF7	E2	421	35	-15.06	3.7	
MCF7	E2+TOT	511	62	-12.42	2.57	
MCF7	E2+TOT	62	2	-3.67	6.64	
MCF7	E2+TOT+IL1b	469	62	-10.19	2.36	
MCF7	E2+TOT+IL1b	495	57	-12.87	2.7	
MCF7	E2+TOT+TNFa	471	50	-13.45	2.92	
MCF7	E2+TOT+TNFa	404	56	-8.26	2.25	
LNCAP	TNF-alpha (1000 U/ml, 2h)	148	47	-0.26	1.1	
LNCAP	TNF-alpha (1000 U/ml, 2h)	158	47	-0.49	1.18	
LNCAP	DHT (100nM, 2h) + TNF-alpha (1000U/ml, 2h)	161	49	-0.42	1.15	

LNCAP	DHT (100nM, 2h) + TNF-alpha (1000U/ml, 2h)	175	59	-0.11	1.04	
LNCAP	DHT (100nM, 2h)	64	8	-2.22	2.57	
LNCAP	DHT (100nM, 2h)	65	14	-0.94	1.57	

Table S8. Promoters of induced genes are involved, in basal condition, in higher numbers of chromatin interactions.

Cell line	Treatment	Data type	Mean number of promoter interactions in the positive set	Mean number of promoter interactions in random sets	p-val
HUVEC	IFN	Hi-C	1.46	0.92	<10E-04
HUVEC	TNFa	Hi-C	1.57	0.95	<10E-04
K562	SAHA	ChIA-PET	5.44	3.34	<10E-04
K562	SAHA	Hi-C	1.12	0.91	<10E-04
K562	NaBut	ChIA-PET	5.27	3.37	<10E-04
K562	NaBut	Hi-C	1.03	0.92	0.0047
HMEC	TNFa	Hi-C	1.4	0.86	<10E-04
MCF7	IL1B	ChIA-PET	7.91	4.52	<10E-04
MCF7	E2 + ICI	ChIA-PET	4.75	4.53	0.41
MCF7	E2+TNFa	ChIA-PET	7.16	4.53	<10E-04
MCF7	E2	ChIA-PET	4.94	4.53	0.04
MCF7	E2+IL1b	ChIA-PET	7.51	4.52	<10E-04
MCF7	E2	ChIA-PET	8.16	4.53	<10E-04
MCF7	IL1b+ICI	ChIA-PET	7.67	4.52	<10E-04
MCF7	TNFa	ChIA-PET	7.27	4.52	<10E-04
MCF7	E2	ChIA-PET	10.63	4.53	<10E-04
MCF7	TNFa+ICI	ChIA-PET	6.89	4.53	<10E-04

IMR90	Nutlin-3a	Hi-C	1.21	0.86	<10E-04
IMR90	TNFa	Hi-C	1.25	0.86	<10E-04
IMR90	TNFa+cyclohex imide	Hi-C	1.24	0.86	<10E-04
GM12878	TNFa	ChIA-PET	3.91	1.81	<10E-04
GM12878	TNFa	Hi-C	1.77	1.27	<10E-04

Table S9. Preference of cell-type specific induced genes to cell-type specific A compartment

HMEC - MCF7									
	AA	AB	BA	BB	total	R	Enrichment		p-value
only induced in cell line 1	29	20	6	9	64	3.44	2.0		0.000952
only induced in cell line 2	267	22	36	33	358	1.67			
induced in both	34	4	1	4	43				
IMR90 - MCF7									
	AA	AB	BA	BB	total	R	Enrichment		p-value
only induced in cell line 1	312	58	65	26	461	0.93	1.52		0.000115
only induced in cell line 2	193	19	70	30	312	3.67			
induced in both	62	7	13	7	89				
GM12878 - IMR90									
	AA	AB	BA	BB	total	R	Enrichment		
only induced in cell line 1	3160	555	130	298	4143	4.33	3.21		
only induced in cell line 2	280	55	38	19	392	0.71			1.49E-06
induced in both	121	30	0	7	158				
GM12878 - MCF7									
	AA	AB	BA	BB	total	R	Enrichment		p-value
only induced in cell line 1	3315	443	243	187	4188	1.83	1.78		0.003586
only induced in cell line 2	213	22	28	25	288	1.25			
induced in both	92	16	5	0	113				

HUVEC - MCF7									
	AA	AB	BA	BB	total	R	Enrichment		p-value
only induced in cell line 1	28	20	2	2	52	9.5	2.0		7.34E-05
only induced in cell line 2	273	26	36	31	366	1.43			
induced in both	27	3	2	3	35				
HMEC - GM12878									
	AA	AB	BA	BB	total	R	Enrichment		p-value
only induced in cell line 1	35	17	7	6	65	2.36	3.4		7.44E-08
only induced in cell line 2	3437	112	388	322	4259	3			
induced in both	35	0	6	1	42				
HUVEC - GM12878									
	AA	AB	BA	BB	total	R	Enrichment		p-value
only induced in cell line 1	33	16	4	1	54	4.29	1.64		0.000288
only induced in cell line 2	3621	136	213	298	4268	1.67			
induced in both	29	0	3	1	33				
HMEC - IMR90									
	AA	AB	BA	BB	total	R	Enrichment		p-value
only induced in cell line 1	24	9	3	7	43	3	0.67		0.471484
only induced in cell line 2	354	60	33	39	486	0.58			
induced in both	46	8	6	4	64				

HUVEC - IMR90									
	AA	AB	BA	BB	total	R	Enrichment		p-value
only induced in cell line 1	31	12	1	3	47	13	0.47		0.019836
only induced in cell line 2	385	69	21	35	510	0.29			
induced in both	32	3	1	4	40				
HUVEC - HMEC									
	AA	AB	BA	BB	total	R	Enrichment		p-value
only induced in cell line 1	38	11	2	2	53	5.25	1.67		0.236958
only induced in cell line 2	59	7	4	3	73	0.5			
induced in both	24	5	0	5	34				

References

1. Barutcu AR, Lajoie BR, McCord RP, Tye CE, Hong D, Messier TL, Browne G, van Wijnen AJ, Lian JB, Stein JL, et al: **Chromatin interaction analysis reveals changes in small chromosome and telomere clustering between epithelial and breast cancer cells.** *Genome Biol* 2015, **16:**214.
2. Taberlay PC, Achinger-Kawecka J, Lun AT, Buske FA, Sabir K, Gould CM, Zotenko E, Bert SA, Giles KA, Bauer DC, et al: **Three-dimensional disorganization of the cancer genome occurs coincident with long-range genetic and epigenetic alterations.** *Genome Res* 2016, **26:**719-731.
3. Rao SS, Huntley MH, Durand NC, Stamenova EK, Bochkov ID, Robinson JT, Sanborn AL, Machol I, Omer AD, Lander ES, Aiden EL: **A 3D map of the human genome at kilobase resolution reveals principles of chromatin looping.** *Cell* 2014, **159:**1665-1680.
4. Le Dily F, Bau D, Pohl A, Vicent GP, Serra F, Soronellas D, Castellano G, Wright RH, Ballare C, Filion G, et al: **Distinct structural transitions of chromatin topological domains correlate with coordinated hormone-induced gene regulation.** *Genes Dev* 2014, **28:**2151-2162.
5. Jin F, Li Y, Dixon JR, Selvaraj S, Ye Z, Lee AY, Yen CA, Schmitt AD, Espinoza CA, Ren B: **A high-resolution map of the three-dimensional chromatin interactome in human cells.** *Nature* 2013, **503:**290-294.
6. Malinen M, Niskanen EA, Kaikkonen MU, Palvimo JJ: **Crosstalk between androgen and pro-inflammatory signaling remodels androgen receptor and NF-kappaB cistrome to reprogram the prostate cancer cell transcriptome.** *Nucleic Acids Res* 2017, **45:**619-630.
7. Stender JD, Nwachukwu JC, Kastrati I, Kim Y, Strid T, Yakir M, Srinivasan S, Nowak J, Izard T, Rangarajan ES, et al: **Structural and Molecular Mechanisms of Cytokine-Mediated Endocrine Resistance in Human Breast Cancer Cells.** *Mol Cell* 2017, **65:**1122-1135 e1125.
8. Swinstead EE, Miranda TB, Paakinaho V, Baek S, Goldstein I, Hawkins M, Karpova TS, Ball D, Mazza D, Lavis LD, et al: **Steroid Receptors Reprogram FoxA1 Occupancy through Dynamic Chromatin Transitions.** *Cell* 2016, **165:**593-605.
