# Supplementary material for: Genomic meta-analysis of the interplay between 3D chromatin organization and gene expression programs under basal and stress conditions
Source: Epigenetics Chromatin. 2018 Aug 29;11:49. doi: 10.1186/s13072-018-0220-2 (PMC6114837; doi:10.1186/s13072-018-0220-2)
Supplement: Supplementary file 2 — Additional file 2: Fig. S1. Correlation between the magnitude of the PC that represents A/B compartmentalization and gene expression level. Fig. S2. Association between changes in A/B compartmentalization and differential gene expression between cell types. A. For each pair of cell lines, we examined the difference (fold change) in expression level between genes assigned to the AB and BA sets (for a pair of cell lines 1 and 2, AB: genes located in the A compartments in cell line 1 and in B in cell line 2; BA: genes located in the B compartment in cell line 1 and in A in cell line 2). For 27 out of 28 pairwise comparisons (all except HMEC–NHEK), we observed a highly significant association (FDR ≪ 5%) between differential compartmentalization and expression. (p values calculated using Wilcoxon’s test.) B. Correlation between the change in the magnitude of PC1 and change in gene expression level in the comparison between GM12878 and four other cell lines. Fig. S3. Enrichment of TFBSs in the A compartment. ChIP-seq experiments are sorted by p value, and A-B density factors are represented by bars. Red line indicates p value = 0.01. Shown are experiments in the GM12878 cell line. Similar results were observed for all other cell lines (data not shown). Fig. S4. Preference of cell-type-specific TF binding for cell-type-specific compartments. Preference of cell-type-specific TF binding events to AB genomic regions over BA regions is measured by the AB occupancy enrichment ratio. The compared cell lines and the examined TF are indicated below each bar (cell lines are indicated by the first two letters of their name, e.g., GM = GM12878, HU = HUVEC, K5 = K562). Fig. S5. Enrichment of histone modifications in the A/B compartments. The analysis presented in Fig. S3 for TF binding events is applied here to histone modification peaks. Most modifications showed a significant enrichment for the A compartment. The repressive marks H3K27me3 and H3K9me3 showed markedly lower enrichment [file 13072_2018_220_MOESM2_ESM.pdf]

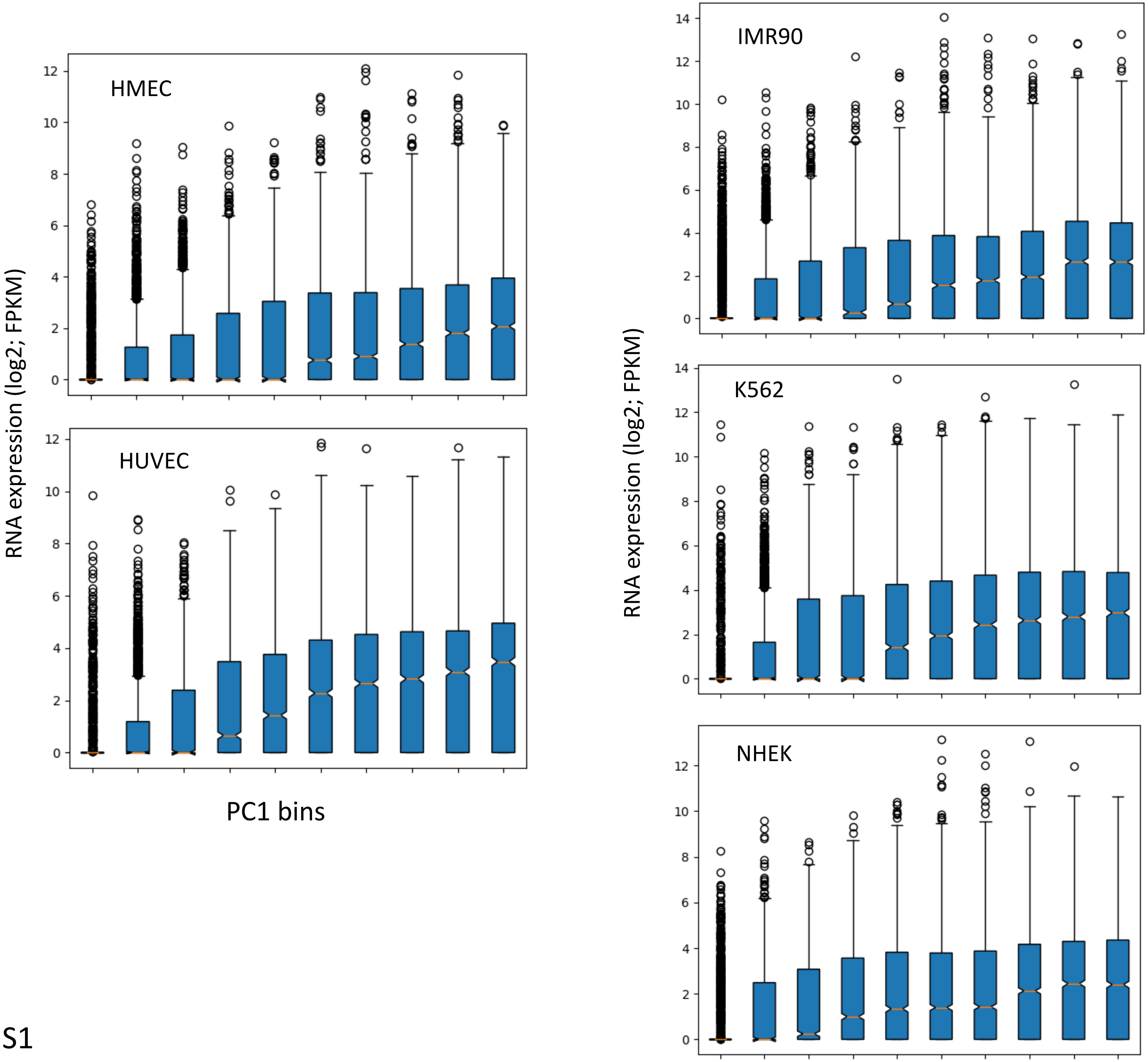

HMEC
HUVEC
IMR90
K562
NHEK
RNA expression (log2; FPKM)
PC1 bins

Fig. S1

A

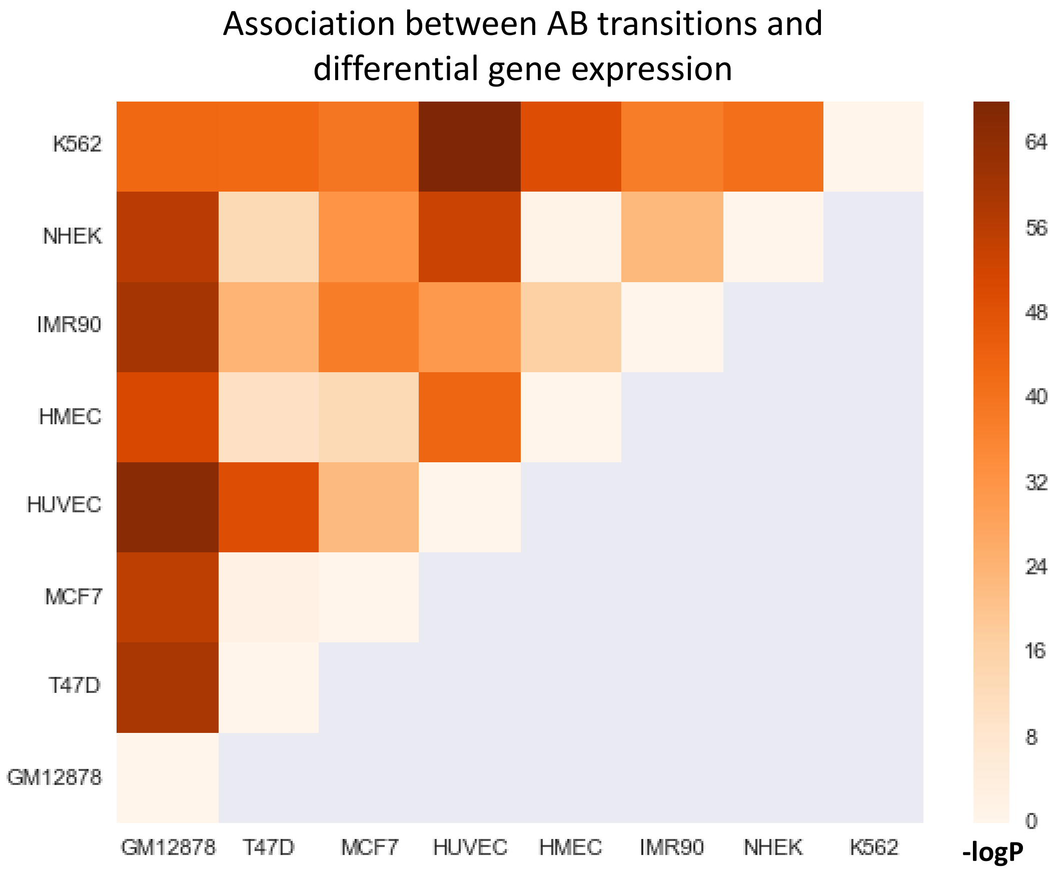
Association between AB transitions and
differential gene expression
K562
NHEK
IMR90
HMEC
HUVEC
MCF7
T47D
GM12878
GM12878
T47D
MCF7
HUVEC
HMEC
IMR90
NHEK
K562
64
56
48
40
32
24
16
8
0
-logP

Fig. S2

B

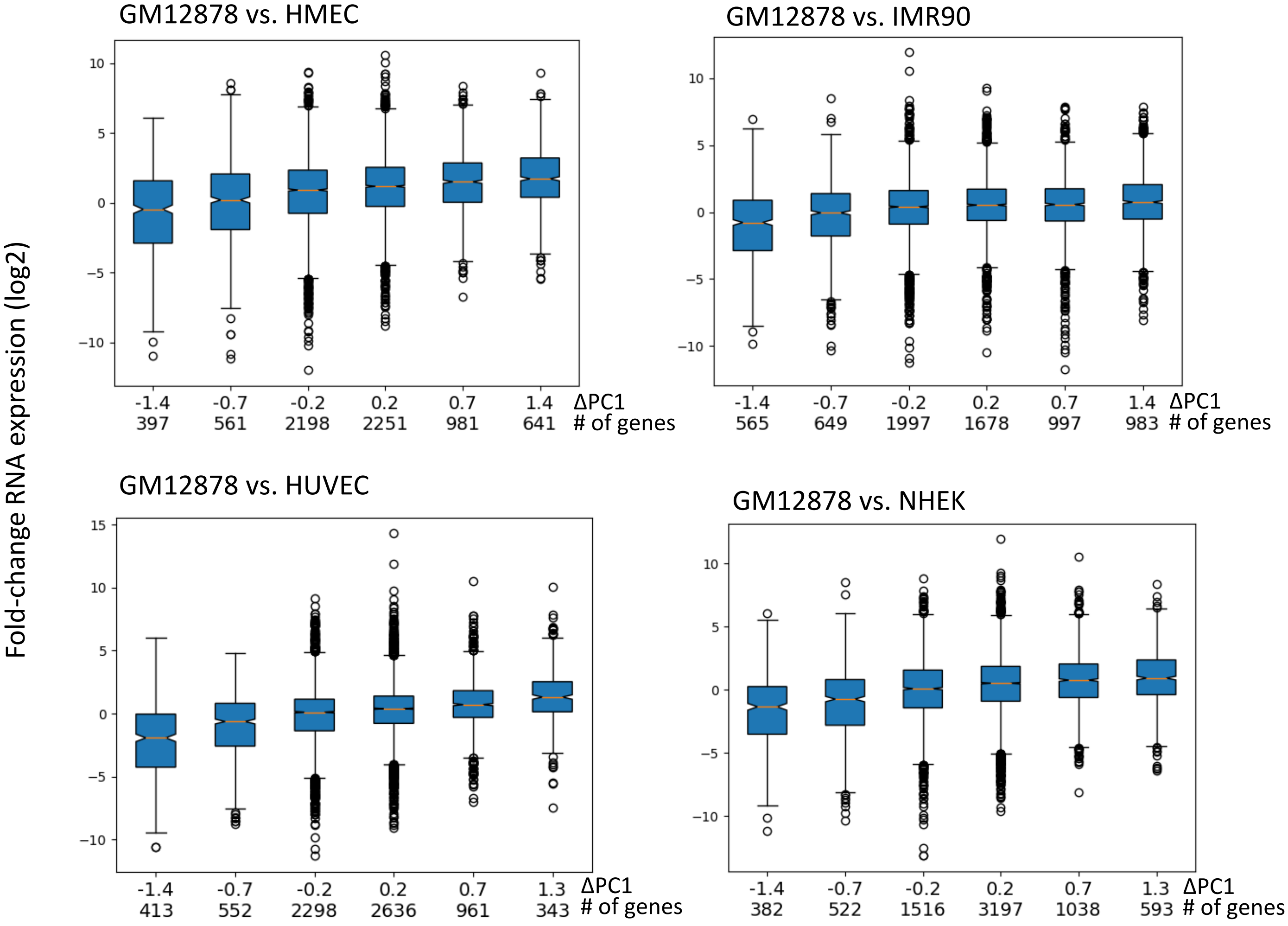

GM12878 vs. HMEC
GM12878 vs. IMR90
GM12878 vs. HUVEC
GM12878 vs. NHEK
Fold-change RNA expression (log2)
-1.4 -0.7 -0.2 0.2 0.7 1.4 ΔPC1
397 561 2198 2251 981 641 # of genes
-1.4 -0.7 -0.2 0.2 0.7 1.4 ΔPC1
565 649 1997 1678 997 983 # of genes
-1.4 -0.7 -0.2 0.2 0.7 1.3 ΔPC1
413 552 2298 2636 961 343 # of genes
-1.4 -0.7 -0.2 0.2 0.7 1.3 ΔPC1
382 522 1516 3197 1038 593 # of genes

Fig. S2

GM12878 – TF enrichment for A compartment

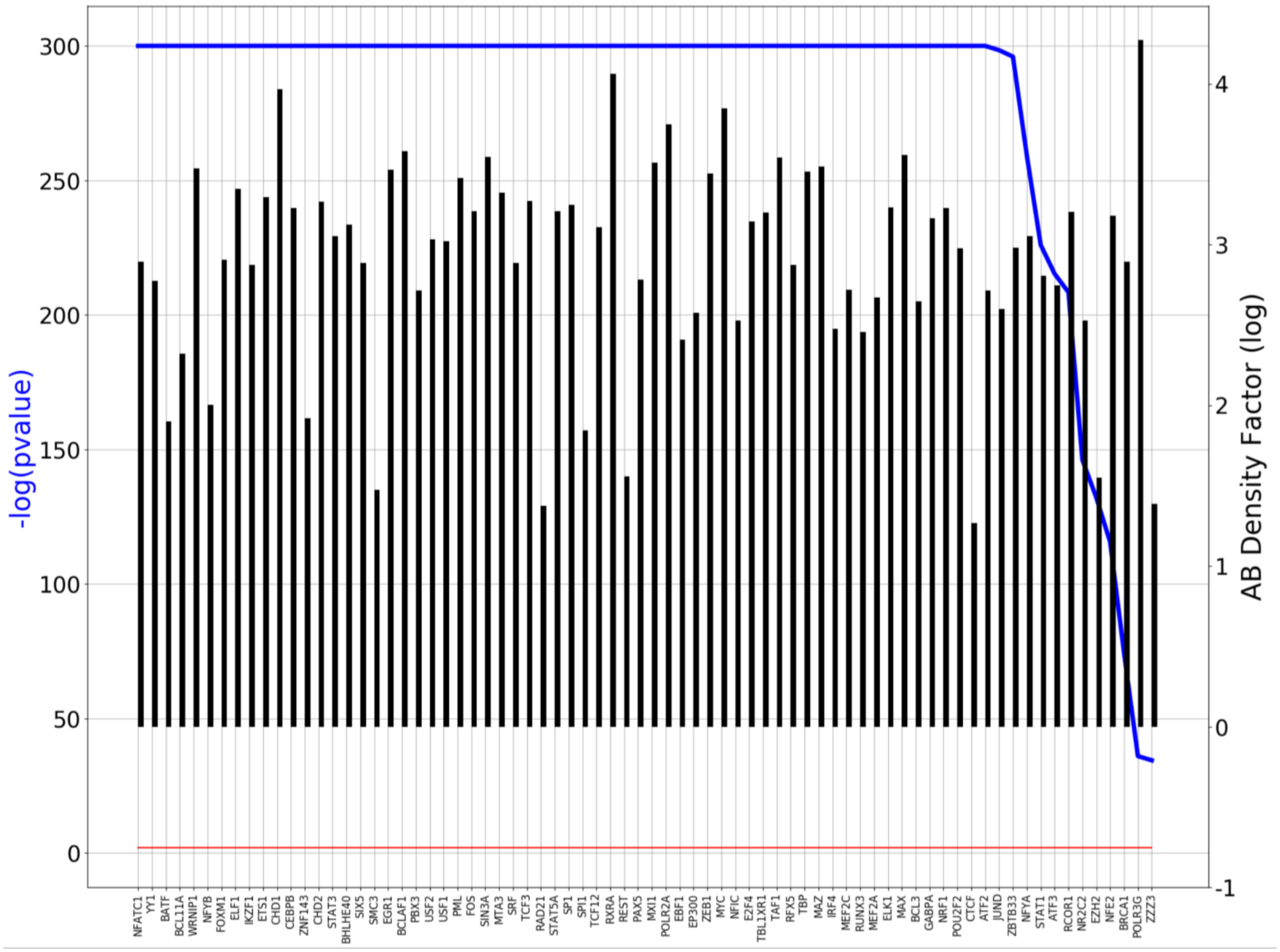

Fig. S3

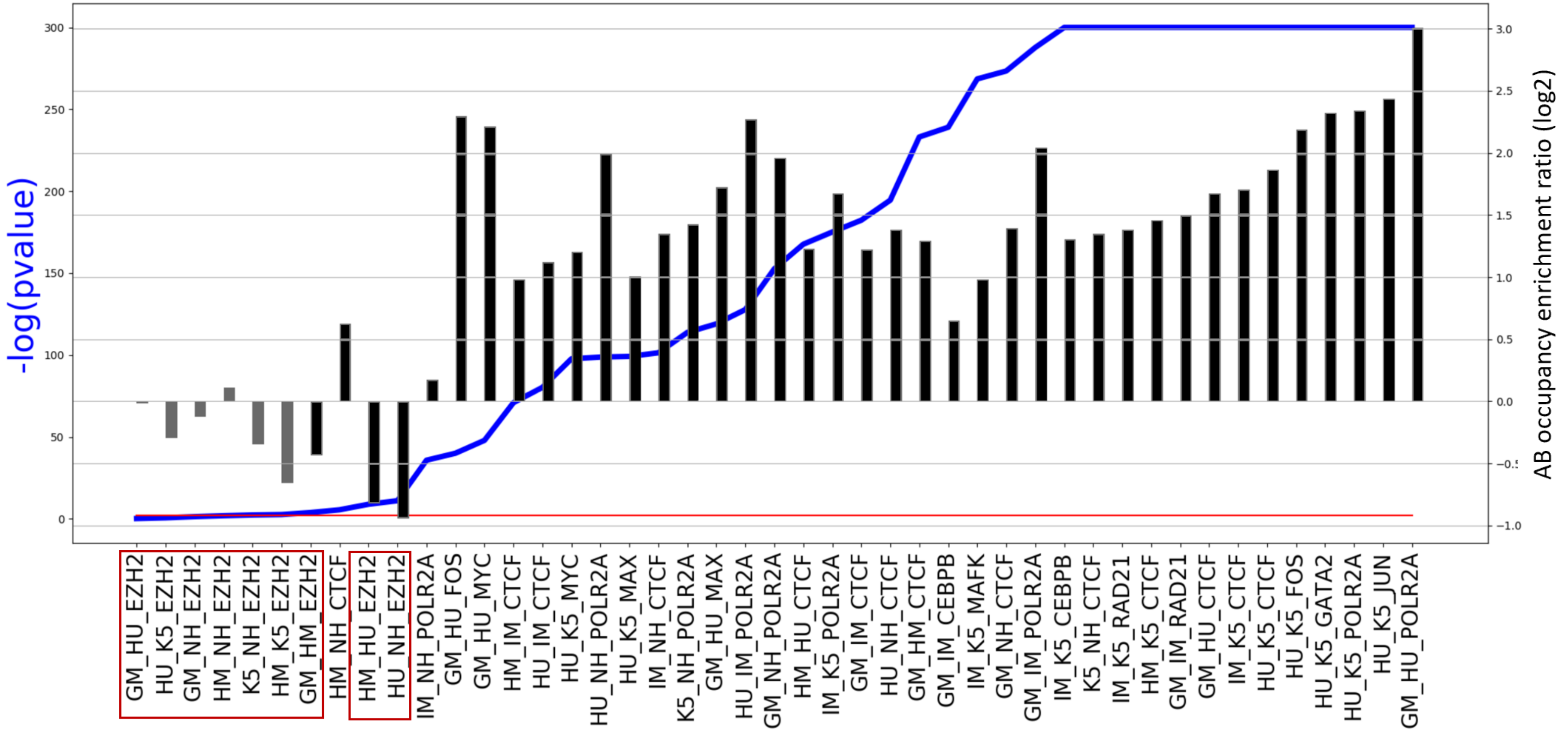
-log(pvalue)
AB occupancy enrichment ratio (log2)
300
250
200
150
100
50
0
3.0
2.5
2.0
1.5
1.0
0.5
0.0
−1.0
GM_HU_EZH2
HU_K5_EZH2
GM_NH_EZH2
HM_NH_EZH2
K5_NH_EZH2
HM_K5_EZH2
GM_HM_EZH2
HM_NH_CTCF
HM_HU_EZH2
HU_NH_EZH2
IM_NH_POLR2A
GM_HU_FOS
GM_HU_MYC
HM_IM_CTCF
HU_IM_CTCF
HU_K5_MYC
HU_NH_POLR2A
HU_K5_MAX
IM_NH_CTCF
K5_NH_POLR2A
GM_HU_MAX
HU_IM_POLR2A
GM_NH_POLR2A
HM_HU_CTCF
IM_K5_POLR2A
GM_IM_CTCF
HU_NH_CTCF
GM_HM_CTCF
GM_IM_CEBPB
IM_K5_MAFK
GM_NH_CTCF
GM_IM_POLR2A
IM_K5_CEBPB
K5_NH_CTCF
IM_K5_RAD21
HM_K5_CTCF
GM_IM_RAD21
GM_HU_CTCF
IM_K5_CTCF
HU_K5_CTCF
HU_K5_FOS
HU_K5_GATA2
HU_K5_POLR2A
HU_K5_JUN
GM_HU_POLR2A

Fig. S4

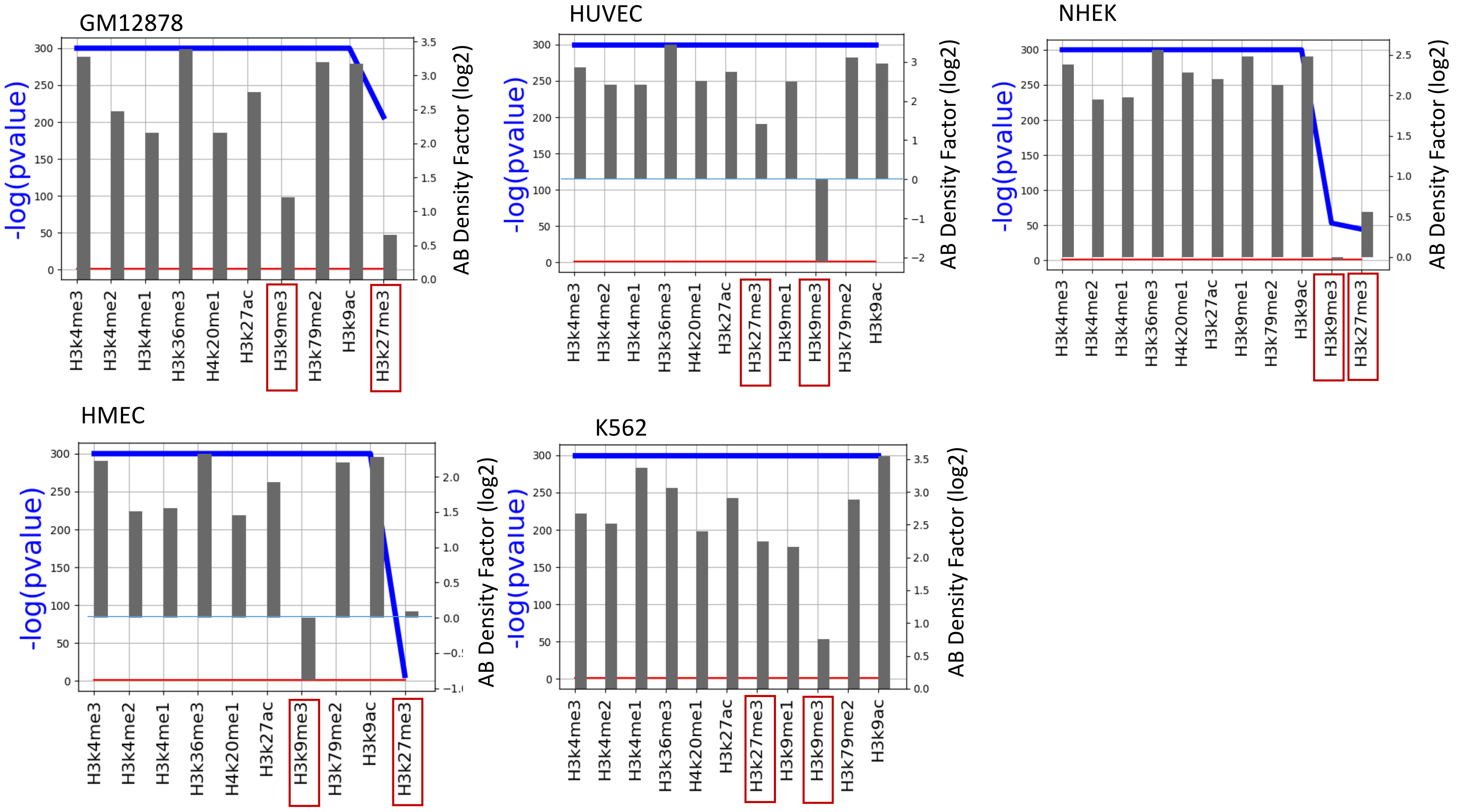
GM12878
HUVEC
NHEK
HMEC
K562
-log(pvalue)
AB Density Factor (log2)
H3k4me3
H3k4me2
H3k4me1
H3k36me3
H4k20me1
H3k27ac
H3k9me3
H3k79me2
H3k9ac
H3k27me3
H3k9me1

Fig. S5

A

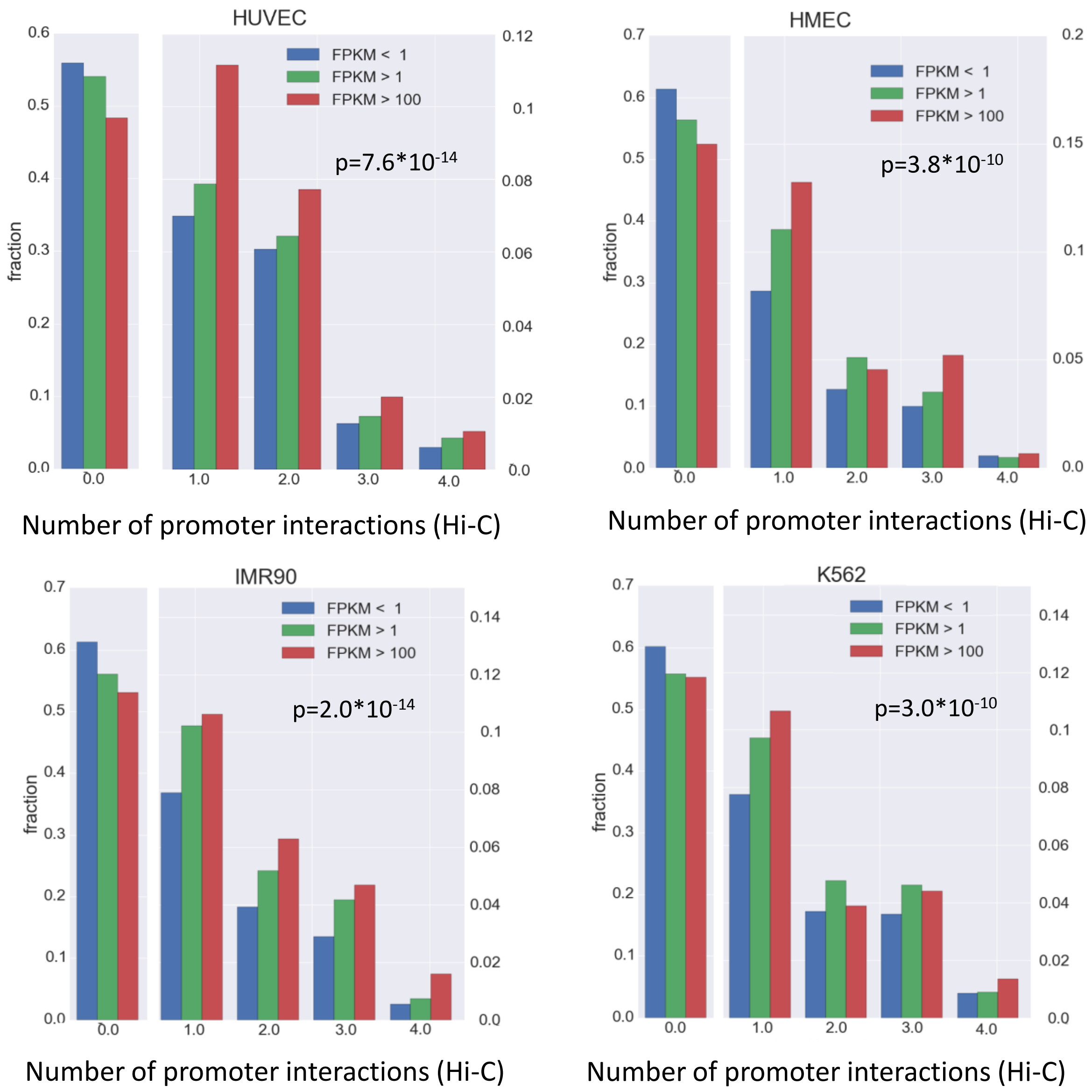
HUVEC
FPKM < 1
FPKM > 1
FPKM > 100
p=7.6*10-14
fraction
Number of promoter interactions (Hi-C)
HMEC
FPKM < 1
FPKM > 1
FPKM > 100
p=3.8*10-10
fraction
Number of promoter interactions (Hi-C)
IMR90
FPKM < 1
FPKM > 1
FPKM > 100
p=2.0*10-14
fraction
Number of promoter interactions (Hi-C)
K562
FPKM < 1
FPKM > 1
FPKM > 100
p=3.0*10-10
fraction
Number of promoter interactions (Hi-C)

Fig. S6

B

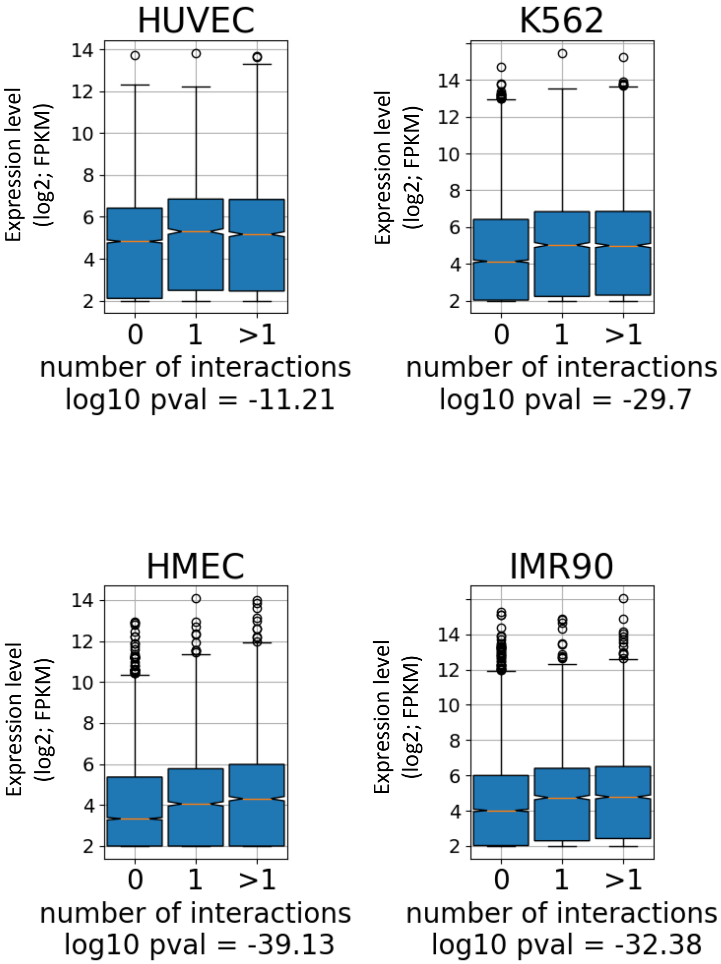
HUVEC
K562
HMEC
IMR90
Expression level
(log2; FPKM)
0
1
>1
number of interactions
log10 pval = -11.21
log10 pval = -29.7
log10 pval = -39.13
log10 pval = -32.38

Fig. S6

C

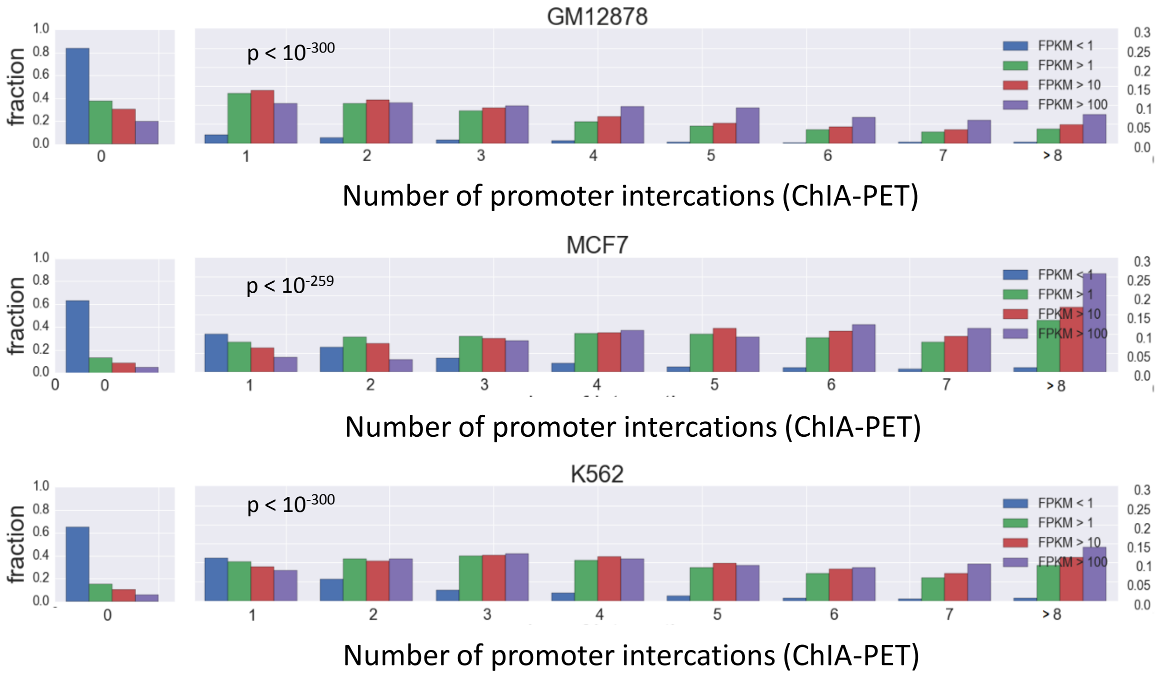

D

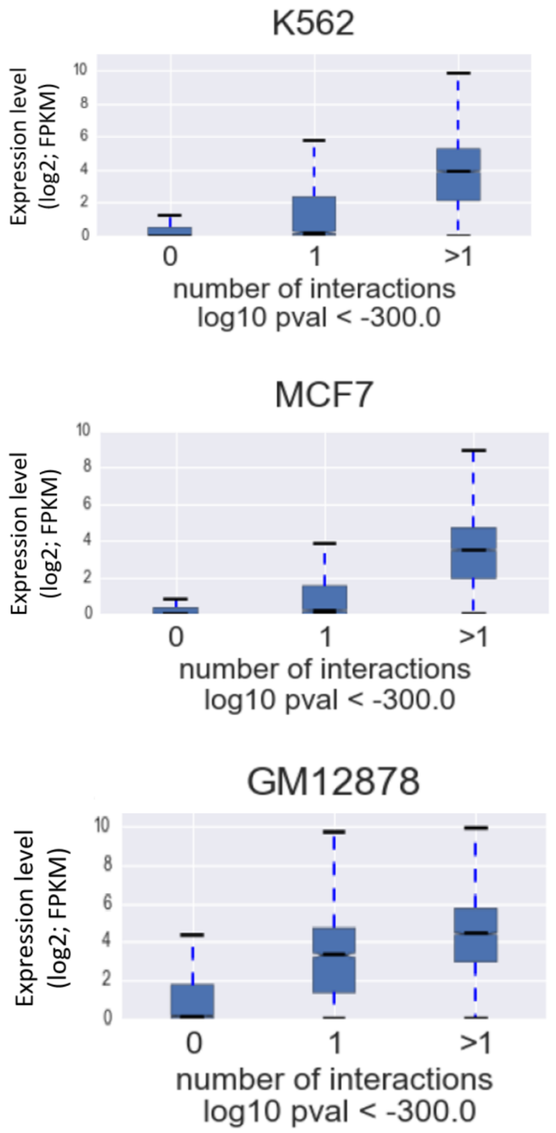

RNA-polII ChIA-PET

Fig. S6

Statistically significant induced genes

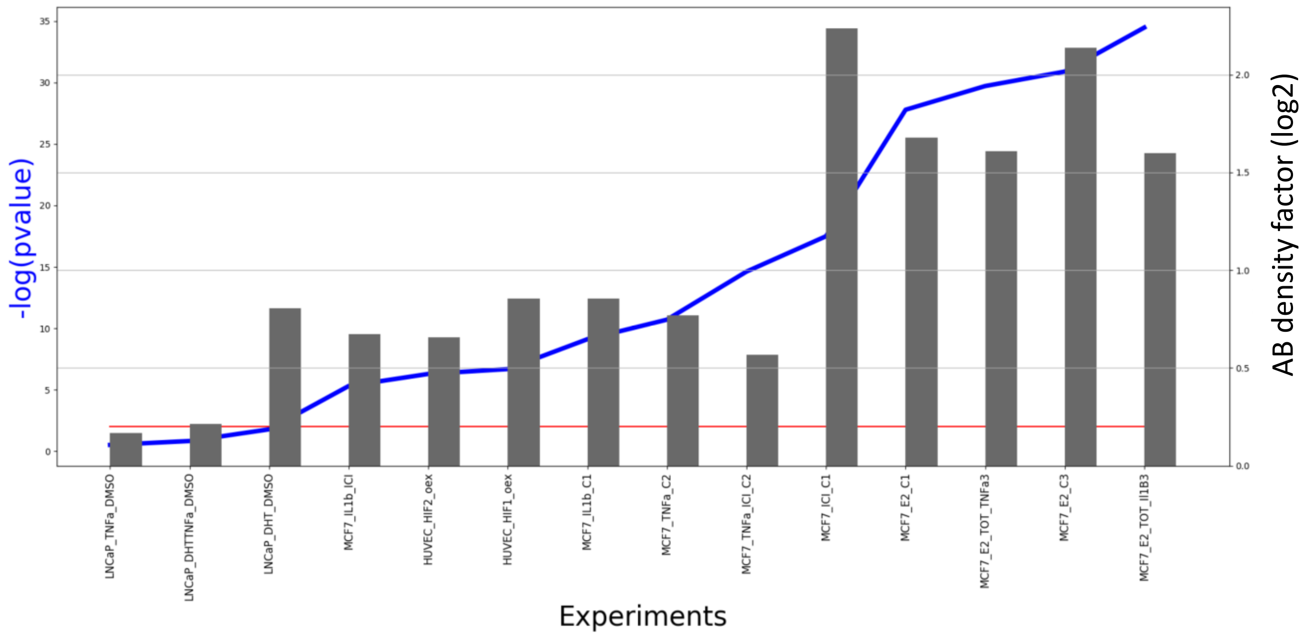

Fig. S7
